# Efficacy and underlying mechanisms of acupuncture therapy for PTSD: evidence from animal and clinical studies

**DOI:** 10.3389/fnbeh.2023.1163718

**Published:** 2023-05-02

**Authors:** Xiaorong Tang, Shumin Lin, Danwei Fang, Binjing Lin, Lulu Yao, Lin Wang, Qin Xu, Liming Lu, Nenggui Xu

**Affiliations:** ^1^South China Research Center for Acupuncture and Moxibustion, Medical College of Acu-Moxi and Rehabilitation, Guangzhou University of Chinese Medicine, Guangzhou, China; ^2^Medical College of Acu-Moxi and Rehabilitation, Guangzhou University of Chinese Medicine, Guangzhou, China; ^3^School of Basic Medical Sciences, Guangzhou University of Chinese Medicine, Guangzhou, China

**Keywords:** acupuncture, posttraumatic stress disorder (PTSD), mechanism, review, animal and clinical studies

## Abstract

As a major public health problem, posttraumatic stress disorder (PTSD) has a substantial impact on individuals and society. The total excess economic burden of PTSD in the US is estimated to be more than $232.2 billion a year. Acupuncture is widely used in patients with PTSD, and an increasing number of studies have been undertaken to assess the efficacy and underlying mechanisms of acupuncture for the treatment of individuals with PTSD. However, there has not yet been a review that simultaneously elucidates the therapeutic efficacy and biological mechanisms of acupuncture. We wished to examine the efficacy and underlying mechanisms of acupuncture for the treatment of individuals with PTSD. We conducted this review in three sections as follows: a meta-analysis, an acupoint analysis, and mechanism research. PubMed, Web of Science, Embase, Cochrane Library, China National Knowledge Infrastructure Database (CNKI), WanFang Database, China Biology Medicine Database (CBM), Chinese Science and Technology Journals Database (VIP), and other databases were searched from 1 January 2012 to 27 November 2022. Based on the included studies, we first determined whether acupuncture is more effective than psychological treatment or pharmacological treatment for treating and improving the quality of life of individuals with PTSD by meta-analysis. Second, the most commonly used acupoints and parameters of acupuncture were summarized based on animal and clinical studies. Third, we attempt to summarize the current mechanisms of acupuncture in the treatment of PTSD. Finally, 56 acupoint analyses, eight meta-analyses, and 33 mechanistic studies were included. Acupuncture outperformed pharmacotherapy treatment in improving symptom scores by CAPS, HAMA, HAMD, PCL-C, and SCL-90 somatization for PTSD and outperformed psychotherapy treatment in improving symptom scores by CAPS PCL-C and HAMD, according to the meta-analysis. GV20 was the most frequently used acupuncture point in clinical studies and animal studies, with a 78.6% application rate. Acupuncture may be effective in treating PTSD by regulating the structure and components of several brain areas, regulating the neuroendocrine system, and involving signaling pathways. In conclusion, this finding indicates that acupuncture has promising potential for treating PTSD.

## 1. Introduction

Post-traumatic stress disorder (PTSD) is a mental disorder that occurs after an individual has experienced intense mental stress, such as an unusual threat or disaster, and lasts for a long time (Battle, 2013). Its main symptoms include reliving the traumatic event, avoiding any thoughts or feelings about the event, having negative feelings about yourself or the world, and having difficulty sleeping or concentrating (Spoont, 2015). According to the World Mental Health Survey Consortium (WMH) survey, 70% of adults in 24 countries have experienced a traumatic event, and 30.5% have been exposed to four or more traumatic events. Of these, 52.5% had been exposed to traumatic events in China (Benjet et al., 2016). According to a World Health Organization (WHO) survey, the lifetime prevalence of PTSD is 3.9%, and it is two times as prevalent in high-income countries as it is in middle-income and low-income countries (Koenen et al., 2017). Treatments available for PTSD span a variety of psychological and pharmacological treatments. Psychological treatment mainly includes prolonged exposure therapy (PE), cognitive processing therapy (CPT), trauma-focused cognitive behavioral therapy (CBT), eye movement desensitization and reprocessing therapy (EMDR), and narrative exposure therapy (NET; Watkins et al., 2018; Guideline Development Panel for the Treatment of PTSD in Adults, 2019). Additionally, paroxetine, fluoxetine, sertraline, venlafaxine, and prazosin are the most commonly used pharmaceutical recommendations (Ursano et al., 2004; Canadian Psychiatric Association, 2006; Wei et al., 2013). However, both psychological treatment and pharmacological treatment are required for the patient's long-term participation. They are time-consuming and costly and have numerous negative consequences, including high treatment-shedding rates and low cure rates (Steenkamp et al., 2015). Furthermore, data show that psychological treatment is primarily dependent on the patient's psychological tolerance and is likely to cause secondary harm to the patient (Lv and Wu, 2022). The most common side effects of pharmacological treatment are gastrointestinal reactions, but psychiatric symptoms, autonomic dysfunction, and extrapyramidal symptoms can also occur (Zhang et al., 2017). Given this limitation, there is an urgent need to find a simple, inexpensive, and effective treatment for PTSD.

Acupuncture has a long history in China. It has the advantages of being simple to perform, having few side effects, and being less restrictive in terms of conditions (Lu et al., 2022). Acupuncture has been shown to have potential benefits for a variety of mental disorders (Errington-Evans, 2012; Lee et al., 2021). A meta-analysis of acupuncture for PTSD in adults found that it can help with PTSD core symptoms and has fewer side effects (Grant et al., 2018). Moreover, animal studies have shown that acupuncture treatment can reduce anxiety, depression, and fear responses; improve sleep architecture; reduce spatial learning and memory deficits; and ultimately improve PTSD (Kwon et al., 2021). Research has shown that (Hollifield, 2011) the mechanism of acupuncture treatment for PTSD may be related to biological mechanisms such as central nervous system improvement and hypothalamic–pituitary–adrenal axis (HPA) regulation. The mechanism of acupuncture treatment for PTSD may involve the regulation of neural circuits, neurotransmitter and receptor expression, signaling pathways, apoptosis, immune cytokines, and the endocannabinoid system (Li et al., 2021). However, to date, there has been no comprehensive review that elucidates both the therapeutic effects and underlying biological mechanisms of acupuncture. To address this, we conducted a systematic review of the evidence-based data on acupuncture treatment for PTSD by searching relevant databases for both efficacy and underlying mechanisms. Specifically, a meta-analysis was used to assess the efficacy of acupuncture for PTSD, and the most commonly used acupoint and parameters of acupuncture were summarized based on animal and clinical studies. In addition, we summarized the current mechanisms of acupuncture in the treatment of PTSD.

## 2. Methods

### 2.1. Search strategy

Two independent individuals searched PubMed, Web of Science, Embase, Cochrane Library, China National Knowledge Infrastructure Database (CNKI), WanFang Database, China Biology Medicine Database (CBM), Chinese Science and Technology Journals Database (VIP), and other databases using the subject terms “posttraumatic stress disorder” and “acupuncture” (detail in Supplementary Table 1). The search period was from 1 January 2012 to 27 November 2022 in both Chinese and English. The protocol of this study was registered with the number INPLASY2022120012.

### 2.2. Eligibility criteria

All human and animal studies on the effects of acupuncture on PTSD are included in this review. A randomized controlled trial (RCT) with PTSD patients was required for meta-analysis; animal models were required for part of the mechanism research; and acupoint analysis had no restrictions on the type of study or subject used.

**Figure 1:**
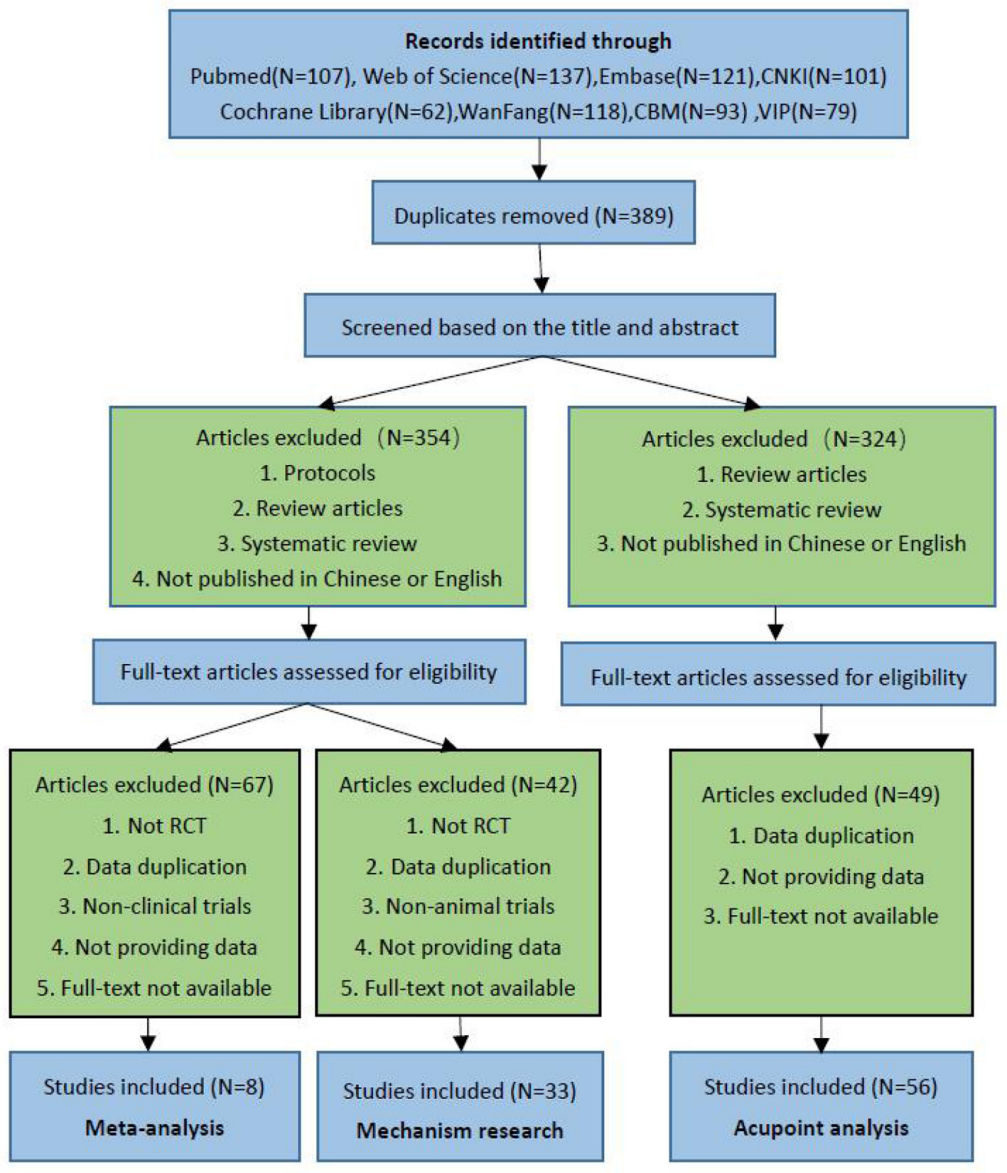
A flow diagram of the literature screening and selection processes.

Screening and evaluation were carried out independently by two independent researchers (Lin SM and Fang DW), with the assistance of a third researcher who intervened if there was disagreement (Lin BJ). Endnote X9 was used to create a database from the included studies. There were 818 studies found during the data search as follows: PubMed (*N* = 107), Web of Science (*N* = 137), Embase (*N* = 121), Cochrane Library (*N* = 62), CNKI (*N* = 101), WanFang (*N* = 118), CBM (*N* = 93), and VIP (*N* = 79). After eliminating 389 duplicates, we read through the titles and abstracts, as well as the full text of the studies, and eventually included eight meta-analyses, 56 acupoint analyses (including 37 animal studies and 19 clinical studies), and 33 mechanistic studies (Figure 1).

### 2.3. Meta-analysis

#### 2.3.1. Selection criteria

We included RCTs for acupuncture in PTSD, with participants divided into acupuncture and non-acupuncture groups. Acupuncture or acupuncture combined with other therapies was available to the acupuncture group, including manual acupuncture (MA), electric acupuncture (EA), and transcutaneous electrical acupoint stimulation (TEAS), while psychological treatment or pharmacological treatment was available to the non-acupuncture group, such as sertraline, paroxetine, and CBT. The outcomes included the Clinician-Administered PTSD scale (CAPS), the PTSD Checklist-Civilian version (PCL-C), the Hamilton Depression Rating Scale (HAMD), the Hamilton Anxiety Rating Scale (HAMA), and the Self-Reporting Inventory 90 somatization (SCL-90 somatization). Patients with major physical and mental illnesses, such as heart, liver, and kidney diseases, were excluded. We also excluded pregnant and lactating women to ensure the accuracy of the experimental data.

#### 2.3.2. Risk of bias assessment

To ascertain the validity of eligible randomized trials, two researchers independently assessed potential risks of bias for all included studies using Cochrane's tool. They assessed all seven domains (random sequence generation, allocation concealment, blinding of participants and personnel, blinding of outcome assessment, incomplete outcome data, selective reporting, and other bias) for each study and assigned a score (high, low, or unclear) depending on their respective judgment.

#### 2.3.3. Statistical synthesis and analysis

For continuous variables, we used both mean differences *(MDs*) and weighted mean differences with 95% *CIs*. RevMan V.5.4 software offered by Cochrane collaboration was used for all data analyses. The meta-analysis followed strict PRISMA guidelines, and tests of heterogeneity were performed using the chi-square test. Fixed effect models were chosen if there was small statistical heterogeneity between the results of these studies (*I*^2^ ≤ 50%). If there was large statistical heterogeneity between studies (*I*^2^ > 50%), random effect models were chosen.

## 3. Results

### 3.1. Meta-analysis

#### 3.1.1. Study description

A total of eight studies (Wang et al., 2012; Wu et al., 2013; Engel et al., 2014; Zhao et al., 2014, 2020; Zhou et al., 2015; Lu et al., 2016; Feng et al., 2019) were included, five in Chinese and three in English. A total of 656 patients with PTSD were enrolled, with 330 in the acupuncture group and 326 in the non-acupuncture group. In the acupuncture group, EA, MA, and TEAS were mainly used. Among them, Wu et al. (2013), Engel et al. (2014), Zhou et al. (2015), and Feng et al. (2019) also combined pharmacotherapy treatment for intervention. In addition, Engel et al. (2014) and Feng et al. (2019) used a combination of acupuncture, pharmacotherapy treatment, and psychotherapy treatment as an intervention in the acupuncture group. In the non-acupuncture group, the main interventions were pharmacotherapy treatment and psychotherapy treatment. The eight articles (Wang et al., 2012; Wu et al., 2013; Engel et al., 2014; Zhao et al., 2014, 2020; Zhou et al., 2015; Lu et al., 2016; Feng et al., 2019) included in this section all used pharmacotherapy treatment, mainly sertraline and paroxetine. Among them, Engel et al. (2014) and Feng et al. (2019) used both pharmacotherapy and psychotherapy in the intervention of the non-acupuncture group (detail in Supplementary Table 2).

#### 3.1.2. Evaluation of methodological quality

The risk of bias in the eight included studies was assessed, and the methodological quality of the clinical trials was generally low (detail in Supplementary Figure 1).

#### 3.1.3. Meta-analysis of outcome measures

##### 3.1.3.1. CAPS

**Figure 2:**
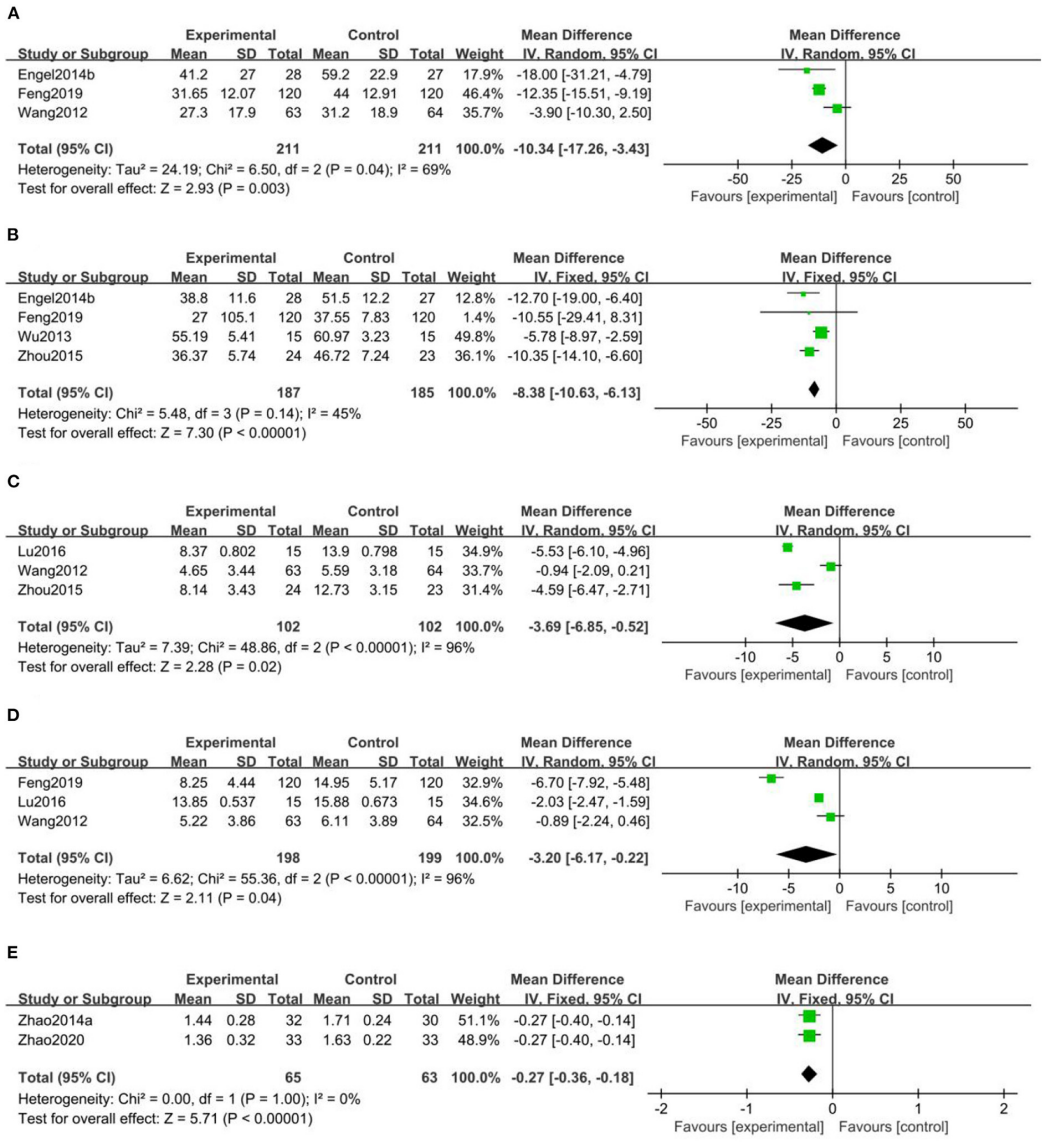
Meta-analysis between acupuncture and pharmacotherapy or psychotherapy treatment. **(A)** The Clinician-Administered PTSD Scale score (CAPS) meta-analysis, **(B)** the PTSD Checklist-Civilian Version score (PCL-C) meta-analysis, **(C)** the Hamilton Depression Rating Scale score (HAMA) meta-analysis, **(D)** the Hamilton Anxiety Rating Scale score (HAMD) meta-analysis, **(E)** the Self-Reporting Inventory 90 somatization score (SCL-90 somatization) meta-analysis.

Three studies including 422 patients conducted a meta-analysis of the CAPS score (Wang et al., 2012; Engel et al., 2014; Feng et al., 2019). Based on heterogeneity results, *I*^2^ = 69%, a random effect model was used. A statistically significant difference in CAPS (*MD* = −10.34, *95% CI* [−17.26, −3.43]) was found between the three groups (*Z* = 2.93, *P* = 0.003), indicating that acupuncture was better than pharmacotherapy or psychotherapy treatment in improving CAPS symptom scores (Figure 2A).

##### 3.1.3.2. PCL-C

Four studies including 372 patients conducted a meta-analysis of the PCL-C score (Wang et al., 2012; Engel et al., 2014; Zhou et al., 2015; Feng et al., 2019). Based on the heterogeneity results, *I*^2^ = 45%, a fixed effect model was used. A statistically significant difference in PCL-C (*MD* = −8.38, *95% CI* [−10.63, −6.13]) was found between the four groups (*Z* = 7.30, *P* < 0.00001), indicating that acupuncture was better than pharmacotherapy or psychotherapy treatment in improving CAPS symptom scores (Figure 2B).

##### 3.1.3.3. HAMA

Three studies including 204 patients conducted a meta-analysis of the HAMA score (Wang et al., 2012; Zhou et al., 2015; Lu et al., 2016). Based on the heterogeneity results, *I*^2^ = 96%, a random effect model was used. A statistically significant difference in HAMA scores (*MD* = −3.*69, 95% CI* [−6.85, −0.52]) was found between the three groups (*Z* = 2.28, *P* = 0.02), indicating that acupuncture was better than pharmacotherapy in improving HAMA symptom scores (Figure 2C).

##### 3.1.3.4. HAMD

Three studies including 397 patients conducted a meta-analysis of HAMD scores (Wang et al., 2012; Lu et al., 2016; Feng et al., 2019). Based on the heterogeneity results, *I*^2^ = 96%, a random effect model was used. A statistically significant difference in HAMD (*MD* = −3.*20, 95% CI* [−6.17, −0.22]) was found between the three groups (*Z* = 2.11, *P* = 0.04), indicating that acupuncture was better than pharmacotherapy or psychotherapy in improving HAMD symptom scores (Figure 2D).

##### 3.1.3.5. SCL-90 somatization

A total of two studies including 128 patients conducted a meta-analysis of the SCL-90 somatization score (Zhao et al., 2014, 2020). Based on heterogeneity results, *I*^2^ = 0%, a fixed effect model was used. A statistically significant difference in HAMD (*MD* = −0.27, *95% CI* [−0.36, −0.18]) was found between the two groups (*Z* = 5.71, *P* < 0.00001), indicating that acupuncture was better than pharmacotherapy in improving SCL-90 somatization symptom scores (Figure 2E).

##### 3.1.3.6. Adverse reactions

During meta-analysis, four studies (Wang et al., 2012; Zhou et al., 2015; Feng et al., 2019; Zhao et al., 2020) reported adverse reactions. The acupuncture group experienced mild needle pain, small amounts of superficial bleeding, small amounts of hematoma, headache, night sweats, nausea, and fatigue. Dizziness, headache, nausea, vomiting, tachycardia, trembling hands, fatigue, depression, decreased appetite, decreased libido, lethargy or insomnia, excessive sedation or agitation, diarrhea or constipation, dry eyes or blurred vision, nasal congestion, and skin irritation were reported in the non-acupuncture group. One of the studies (Feng et al., 2019) found that the pharmacological (sertraline) treatment group had a higher rate of adverse reactions than the non-pharmacological treatment group.

#### 3.1.4. Identify heterogeneity

We have observed a substantial degree of heterogeneity among the studies included in the meta-analysis. As such, we have employed a stepwise exclusion approach to ascertain the origins of this heterogeneity. We discovered that the meta-analysis of the HAMA and HAMD symptom scores had significant heterogeneity. Through the technique of article-by-article elimination, we think Wang et al. (2012) probably may be the sources of heterogeneity in HAMA meta-analysis, while Feng et al. (2019) may be the sources of heterogeneity in HAMD meta-analysis (detail in Supplementary Figures 2, 3). After carefully reading the entire article, heterogeneity may stem from the diversity of intervention measures. Specifically, while other studies mainly used acupuncture as the sole treatment in the acupuncture group, Feng et al. (2019) used acupuncture in combination with CBT and sertraline in the basic treatment. Moreover, other studies mainly used sertraline as a control measure, but Wang et al. (2012) used paroxetine. This may have affected the analysis of the effect of acupuncture in the study and ultimately influenced the results. However, despite this heterogeneity, the overall findings still demonstrate that acupuncture is more efficacious than the control group, thus the current meta-analysis results are still relatively reliable.

#### 3.1.5. Results of the meta-analysis

We discovered that acupuncture significantly improved symptom scores in CAPS, HAMA, HAMD, PCL-C, and SCL-90 somatization compared to pharmacotherapy in the meta-analysis. Moreover, acupuncture also improved symptom scores in CAPS, PCL-C, and HAMD compared to psychotherapy. Furthermore, no serious adverse events related to acupuncture treatment were reported in the studies included in this meta-analysis. Together, this finding indicates that acupuncture has promising potential for treating PTSD.

### 3.2. Acupoint analysis

In the meta-analysis section, acupuncture improved symptom scores in CAPS, HAMA, HAMD, PCL-C, and SCL-90 somatization in PTSD patients. Following the confirmation of acupuncture's efficacy in the treatment of PTSD, we will summarize and analyze the articles on animal and clinical studies of acupuncture in the treatment of PTSD, with a focus on the selection of acupoints and acupuncture parameters.

#### 3.2.1. Study description

A total of 56 studies were included, with 19 clinical studies and 37 animal studies. Two studies (Prisco et al., 2013; King Heather et al., 2015) used ear acupuncture, one study (Wu et al., 2013) used scalp acupuncture, one study (Yang et al., 2022) used CO_2_ laser stimulation, one study (Feng et al., 2019) used TEAS, and other studies (Fang et al., 2012; Li, 2012, 2014, 2018, 2020; Wang et al., 2012, 2015; Hou et al., 2013a,b, 2021; Wang, 2013, 2019; Engel et al., 2014; Li and Zhao, 2014; Zhao et al., 2014, 2019, 2020; Xie et al., 2015; Zheng, 2015; Zheng et al., 2015; Zhou et al., 2015, 2019; Lu et al., 2016; Zhu et al., 2016, 2022; Han, 2017; Li et al., 2017, 2020a,b; Ding, 2018; Oh et al., 2018; Zhao Y. et al., 2018; Zhao Z. et al., 2018; Chen et al., 2019, 2020, 2021; Liu et al., 2019, 2020; Moiraghi et al., 2019; Wei et al., 2019; Xue et al., 2019; Yu et al., 2019; Zhang et al., 2019; Zhu and Lu, 2019; Alvear, 2021; Hollifield et al., 2021; Abanes et al., 2022; Lee and Pan, 2022; Song et al., 2022; Sun Y. et al., 2022; Zhou C. et al., 2022) used filiform needle (detail in Supplementary Tables 3, 4).

#### 3.2.2. Analysis of acupoints

**Figure 3:**
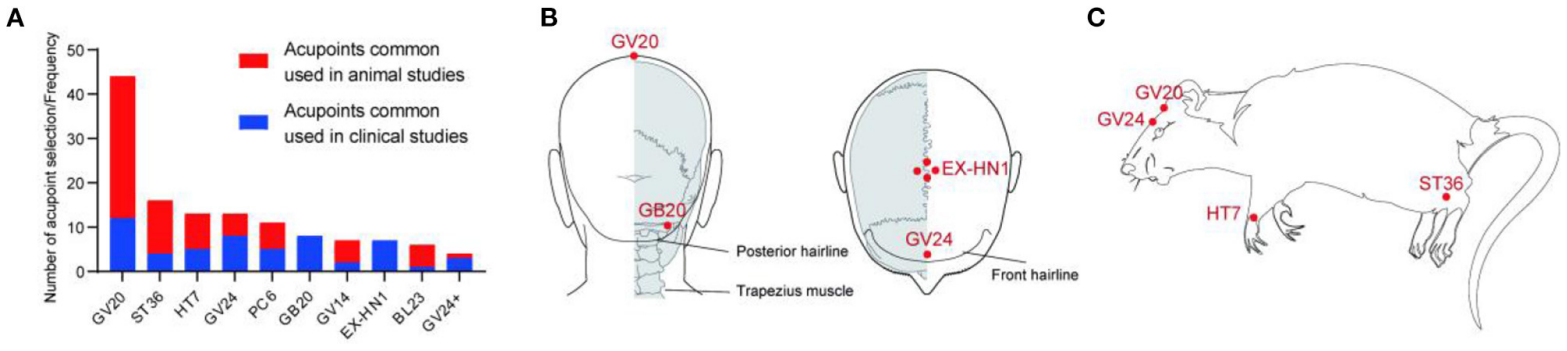
The use frequency of acupoints and location of representative acupoints in animals and humans with PTSD. **(A)** The use frequency of acupoints, including animals and humans with PTSD. **(B)** The most commonly used acupoints for PTSD patients. **(C)** The most commonly used acupoint for PTSD model animals.

The latest standard GB/T 12346-2021 nomenclature and location of meridian points are used for acupoint names and localization in humans in this review, and animals adopt the group standard T/CAAM 0002-2020. Laboratory animals adopt acupoint names and positioning Part 2: Rats and Part 3: Mice. The top four acupoints used in clinical studies were GV20, GV24, GB20, and EX-HN1, and the top four acupoints used in animal studies were GV20, ST36, HT7, and GV24 (Figure 3). Areas of pathological reactions of the body caused by lesions are referred to as acupoints, which are generally a multilevel “three-dimensional structure” composed of nerves, blood vessels, muscles, fascia, tendons, and other tissues, with the function of feeling stimulation and reflecting disease symptoms (Zhu, 2021). GV20 is the most frequently used acupoint, whether in clinical studies or animal studies. GV20 was used in 12 of the 19 clinical studies and 32 of the 37 animal studies. Acupuncture regulates autonomic nerves *via* cerebral cortex reflexes, increases the excitability of relevant parts of the cerebral cortex, and has a therapeutic role in PTSD (Hou et al., 2013a). According to published studies, GV20 can improve neuroinflammation-related cognitive dysfunction and modulate depression and anxiety behavior (Yue et al., 2018; Sun L. et al., 2022). Because PTSD is a mental systemic disease that is closely related to the brain, there is a greater selection of brain acupoints in the treatment of PTSD. Among the top four acupoints, clinical studies had four brain acupuncture points, such as GV20, GV24, GB20, and EX-HN1, and animal studies had two, GV20 and GV24. Compared to clinical studies, animal studies use a simpler selection of acupoints, usually two to four, and this simple selection of acupoints can better reflect the specific role of each acupoint. In clinical studies, acupoint selection is more abundant. The matching relationship between acupoints is considered more, and disease treatment is more comprehensive.

#### 3.2.3. Analysis of acupuncture parameters

Acupuncture prescriptions are generally thought to include acupoints, timing, and acupuncture methods. Acupuncture time was generally chosen for 15, 20, or 30 min per day, for 7, 14, or 21 days in animal studies, with 10 studies (Hou et al., 2013a,b; Xie et al., 2015; Li et al., 2017; Chen et al., 2019, 2020; Xue et al., 2019; Zhou et al., 2019; Zhou Q. et al., 2022; Zhu et al., 2022) choosing 30 min per day for 7 days. Clinical studies are typically 30 min per day, with longer durations of 4, 8, 12 Weeks, and so on. Both electroacupuncture (EA) and manual acupuncture (MA) were used. EA is an acupuncture method that uses a small current close to the body's bioelectricity to change the ion distribution on both sides of the cell membrane and has a great therapeutic effect. Continuous waves of 2 Hz or alternating waves of 2/15 Hz were used frequently in animal studies. Overall, animal studies mostly use low-frequency electroacupuncture. Clinical studies primarily use 100 Hz continuous waves. The different choices for acupuncture frequency may be due to the different effects of different acupuncture frequencies. Acupuncture at the same acupoint with different frequencies can activate different brain regions. Low-frequency electroacupuncture selectively activates the ventral thalamus, hypothalamus, and entorhinal cortex, whereas high-frequency electroacupuncture selectively activates the dorsal thalamus and cingulate gyrus (Jin et al., 2001). Low-frequency electroacupuncture promotes the release of endorphins and enkephalins, high-frequency electroacupuncture promotes the release of dynorphins, and frequency conversion promotes the corelease of enkephalins and dynorphins (Zhao, 2008). Low-frequency electroacupuncture can boost EAAT1 and EAAT2 mRNA expression in the hippocampus, improve glutamate recycling, and provide antidepressant effects (Lu et al., 2003; Ji et al., 2013). High-frequency electroacupuncture has a better neuroprotective effect, as it can lower malondialdehyde levels and boost superoxide dismutase activity, reducing free radical damage, protecting nerve cells, and repairing damaged tissues (Shi et al., 2017). We believe that physiological differences between humans and animals may influence acupuncture frequency selection. PTSD animal models frequently select rodents, which have very different bodies from humans. The stimulation parameters should reflect the amount of stimulation received rather than the amount of stimulation applied, and judging the actual amount of electroacupuncture effect received by animals is difficult. Furthermore, animals mostly receive acupuncture while under anesthesia, whereas patients receive acupuncture while awake, which may have an impact on acupuncture translational medicine (Zhou C. et al., 2022).

### 3.3. Mechanism research

In the above review, we have learned about the role of acupuncture in the treatment of PTSD and the choice of common acupoints and parameters for acupuncture. Next, we will summarize the mechanisms or specific cellular and molecular events of acupuncture in the treatment of PTSD as much as possible.

#### 3.3.1. Study description

A total of 33 studies were included. Except for four studies (Wang, 2019; Chen et al., 2021; Zhou C. et al., 2022; Zhu et al., 2022) that used C57BL/6 mice, all of the remaining studies used rats. Establishing a good model of PTSD is critical to studying its pathogenesis (Pitman et al., 2012). Most of the studies (Yang and Zhang, 2012; Hou et al., 2013a,b; Xie et al., 2015; Zhu et al., 2016, 2022; Li et al., 2017, 2020a,b; Ding, 2018; Oh et al., 2018; Feng et al., 2019; Liu et al., 2019, 2020; Wang, 2019; Wei et al., 2019; Zhu and Lu, 2019; Li, 2020; Lee and Pan, 2022; Sun Y. et al., 2022; Yang et al., 2022; Zhou C. et al., 2022) included in this section used single prolonged stress (SPS) (Liberzon et al., 1997), five studies (Zhao Y. et al., 2018; Zhao Z. et al., 2018; Yu et al., 2019; Zhang et al., 2019; Zhao et al., 2019) used shock and claustrophobia, four studies (Li and Zhao, 2014; Chen et al., 2019, 2020; Zhou et al., 2019) used enhanced single prolonged stress (ESPS), two studies (Hou et al., 2021; Zhou C. et al., 2022) used modified single prolonged stress (MSPS), and one study (Chen et al., 2021) used repeated social defeat stress (RSDS). The 33 included studies primarily used the open field test (Xie et al., 2015; Zhu et al., 2016, 2022; Li et al., 2017; Chen et al., 2019, 2020; Liu et al., 2019, 2020; Zhou et al., 2019; Hou et al., 2021; Zhou C. et al., 2022), elevated plus maze (Fang et al., 2012; Li et al., 2017; Chen et al., 2019, 2020; Liu et al., 2019; Zhou et al., 2019; Li, 2020; Hou et al., 2021; Sun Y. et al., 2022; Yang et al., 2022; Zhu et al., 2022), fear conditioning test (Ding, 2018; Zhou et al., 2019; Li et al., 2020a,b; Lee and Pan, 2022; Zhu et al., 2022), Morris water maze (Hou et al., 2013b; Li and Zhao, 2014; Zhao Y. et al., 2018), novel inhibition of feeding (Sun Y. et al., 2022), object recognition task (Zhao Y. et al., 2018; Lee and Pan, 2022), and forced swimming test (Oh et al., 2018). These behavioral experiments were used to identify PTSD-related symptoms, such as anxiety, depression, and sleep disorders, in PTSD model animals (detail in Supplementary Table 5).

#### 3.3.2. Proposed mechanisms

##### 3.3.2.1. The regulation of structure and components in several brain areas

(1) Hippocampus: The hippocampus is a high-level regulator of stress responses and one of the most stress-sensitive brain regions (Zhang et al., 2015). The pathogenesis of PTSD has been linked to abnormalities in the functional structure of the hippocampus (Bremner et al., 2003). The hippocampal volume of PTSD model animals decreased significantly, neuronal apoptosis increased (Li et al., 2007), and negative stress feedback decreased or disappeared (Zhao Z. et al., 2018). Stress causes a neuroinflammatory response by inducing the activation of microglia and astrocytes, resulting in the release of a large number of proinflammatory cytokines. A study demonstrated that acupuncture reduced the expression of activated microglia (Li, 2020), reduced lipocalin-2 in the hippocampus in PTSD model animals (Chen et al., 2021), and inhibited astrocyte activation. Another study showed that acupuncture improved anxiety, learning, and memory in a model by inhibiting the overexpression of key molecules of hippocampal endoplasmic reticulum stress as follows: GRP78, CHOP, Caspase-3, and Caspase-12 (Sun Y. et al., 2022). The Bcl-2 gene is an important antiapoptotic gene that directly inhibits the proapoptotic factor Bax. The Bcl-2/Bax protein effectively regulates the release of the apoptosis-initiating factor cyt-c, activates caspase-3, and mediates cell survival or death (Dong and Gao, 2012). In a study, acupuncture reduced the expression of Bcl-2/Bax protein in the hippocampal CA1 region (Liu et al., 2020), downregulated the expression of COX-2 (Lee and Pan, 2022), and inhibited apoptosis.

Two of the primary brain regions affected by PTSD are the CA1 and CA3 regions. They are mainly involved in the formation of episodic memory for events and environments, which is closely related to cognition (Webler et al., 2019). A recent study revealed that the interspike interval (ISI) was prolonged in the CAI and CA3 regions, and the concentrated PSD distribution area moved down in PTSD (Zhao et al., 2019). In the PTSD model animals, the abnormality of the spatiotemporal pattern of hippocampal CA1 and CA3 regions suggested abnormal neural electrical activity. A similar study demonstrated that, in addition to abnormal neural electrical activity, the CA1 and CA3 regions had varying degrees of damage to ribosomes, organelles, mitochondria, and rough endoplasmic reticulum and that acupuncture can reverse this abnormal neuroelectric activity (Zhao Z. et al., 2018) and restore neuronal cell ultrastructure (Yu et al., 2019).

(2) Amygdala: The amygdala not only plays an important role in the limbic system but also greatly influences the behavior of PTSD patients. Its activity is related to the severity of PTSD, which is primarily manifested in anxiety and fear memory formation (Shin et al., 2004). In a study, acupuncture was shown to increase the expression of amygdala BDNF and weaken TH in PTSD model animals, implying that acupuncture promotes neuroprotection and inhibits the active amygdala, which influences PTSD prevention and treatment (Zhu and Lu, 2019).

(3) Prefrontal cortex (PFC): The “prefrontal-amygdala-hippocampal circuit hypothesis” is another influential PTSD mechanism. The PFC is the high-level regulatory center of fear emotion regulation and the neural basis of cognition (Zhu et al., 2016). If the PFC is damaged, fear memories are difficult to overcome, and PTSD is difficult to treat. Acupuncture was reported to counteract the neurotoxicity of excitatory amino acids, reduce stress-induced nerve cell damage, and promote neuroprotective effects and fear memory regression by upregulating BDNF in the PFC (Chen et al., 2020) and downregulating mPFC GABAARal (Zhu et al., 2016). Another study revealed that IL-6 levels were inversely related to cognitive levels (Gruol, 2015), and acupuncture could reduce IL-6 levels in the PFC (Chen et al., 2020) and reduce anxiety and depression in PTSD model animals. The ventromedial prefrontal cortex (vmPFC) is associated with the regulation of emotional processes, attention, and executive function. A study found that acupuncture increased the expression of C-Fos in the vmPFC in PTSD model animals and that vmPFC inactivation eliminated the effect of acupuncture on anxiety-like behavior in PTSD model animals, indicating that the vmPFC is involved in the process of electro-targeting PTSD anxiety (Hou et al., 2021; Yang et al., 2022).

(4) Anterior cingulate cortex (ACC): The anterior cingulate cortex is a major component of the limbic system, which is involved in emotional and painful processes, and modulates stress and fear responses in PTSD (Jatzko et al., 2013). C-Fos is a gene that promotes cell proliferation and differentiation in response to external stimulation and is a sensitive indicator of neuronal activation (Velazquez et al., 2015). Research has shown that acupuncture increases C-Fos expression in the ACC and decreases anxious behaviors in PTSD model animals (Liu et al., 2019).

(5) Hypothalamus: The hypothalamus is the higher regulatory center in the autonomic nervous system and is an important junction between the limbic system and reticular structures. The PTSD model animals were devoid of sleep-wake behavior, and the sleep times in SWS1 and REMS were significantly reduced. Acupuncture was also reported to improve sleep disorders in PTSD insomnia rats by increasing the TNF content of the hypothalamus and decreasing IL4 expression (Wei et al., 2019).

##### 3.3.2.2. The regulation of PTSD in the neuroendocrine system

(1) Hypothalamus–pituitary–adrenocortical (HPA) axis: The HPA axis is an important part of the neuroendocrine system, regulating and controlling the body's response to stress. Under stress, the hypothalamus secretes corticotropin-releasing hormone (CRH), which acts on the anterior pituitary gland to produce adrenocorticotropin (ACTH). ACTH stimulates the adrenal cortex to produce glucocorticoids (GCs), which include cortisol in humans and corticosterone (CORT) in rodents. Oversecreted GCs activate the hypothalamic-pituitary gland's negative feedback pathway, inhibit the release of ACTH and CRH, and thus regulate the activity of the HPA axis. According to some studies, the pathological basis of PTSD is the inhibition of negative feedback on the HPA axis (Burbiel, 2015). The excessive release of proinflammatory cytokines through the neuroinflammatory response leads to excessive secretion of CRH and CORT (Michopoulos et al., 2015), and excessive CORT will convert short-term fear memories into long-term fear memories that are difficult to forget (de Quervain et al., 2017). A study demonstrated that acupuncture was found to reverse increased levels of the CRH and CRHR1 proteins in PTSD model animals (Zhu et al., 2022). Acupuncture reduced CORT in PTSD model animals and modulated abnormal HPA axis hyperactivity in other studies (Fang et al., 2012; Lee and Pan, 2022). GCs can interact with the glucocorticoid receptor (GR) and the mineralocorticoid receptor (MR). A similar study found that acupuncture increased hippocampal MR expression in PTSD model animals while decreasing GR expression, increasing the MR/GR ratio (Hou et al., 2013b; Wang, 2019), implying that acupuncture may regulate damaged PTSD model hippocampal neurons, regulate the expression of MR and GR, and then affect the activity of the HPA axis.

(2) Neural nitric oxide synthase (nNOS): Nitric oxide (NO) is a small gas molecule with high-fat solubility that can travel quickly within and between cells, where it participates in neuronal growth and development, degenerative changes, and apoptosis. Because NO has a very short half-life, nitric oxide synthase (NOS) is commonly used to react to NO levels indirectly. Previous research has shown that NO can contribute to memory formation through long-term potentiation (LTP) (Takahashi and Okada, 2011). Stress causes the body to release a large amount of NO and promotes the formation of fear memory. Reliving the traumatic event is one of the core symptoms of PTSD, and each reliving is a strong stress response, so the body releases NO again in large quantities, damaging related neurons, and promoting the continuous reinforcement of fear memories. Many studies have shown that acupuncture can reverse the overexpression of nNOS in the hippocampal CA1 and CA3 regions and locus caeruleus, as well as promote fear memory extinction (Hou et al., 2013a; Xie et al., 2015).

(3) Endocannabinoid (eCB): The eCB system is one of the key molecular systems in the central nervous system that regulates synaptic plasticity. The eCB system is involved in the injury response, anxiety, cognition, stress response, and social–behavioral processes. It is primarily made up of cannabinoid receptor types 1 (CB1R) and 2 (CB2R), as well as 2-arachidonoylglycerol (2-AG) and anandamide (AEA) ligands. 2-AG is primarily synthesized by diacylglycerol (DAGL) and degraded by monoacylglycerol (MAGL; Yao and Fang, 2019). CB1R and DAGL protein expression is linked to fear and anxiety behavior. A recent study revealed that acupuncture increases DAGL and CB1R protein expression to improve anxiety behavior in PTSD model animals, implying that the eCB system may be a target for PTSD treatment (Chen et al., 2019; Xue et al., 2019).

(4) Sirt1/MAO-A: 5-HT is primarily found in vesicles at the ends of 5-HT neurons, and current research on 5-HT targets for the treatment of PTSD has focused on selective serotonin reuptake inhibitors (SSRIs) and monoamine oxidase inhibitors (MAOIs; Auxéméry, 2012). There are two types of MAOI as follows: MAO-A and MAO-B. MAO-A is a vital enzyme that controls the levels of 5-HT in the brain, and the deacetylase Sirt1 is a crucial regulator of MAO-A gene transcription. The brain's MAO-A activity is closely linked to the production of anxiety and fear. Overexpression of the deacetylase Sirt1 activates MAO-A and decreases intracerebral 5-HT levels in PTSD model animals, but acupuncture was also reported to reverse this trend and alleviate anxiety behavior (Li et al., 2017).

##### 3.3.2.3. Signaling pathway involvement

**Figure 4:**
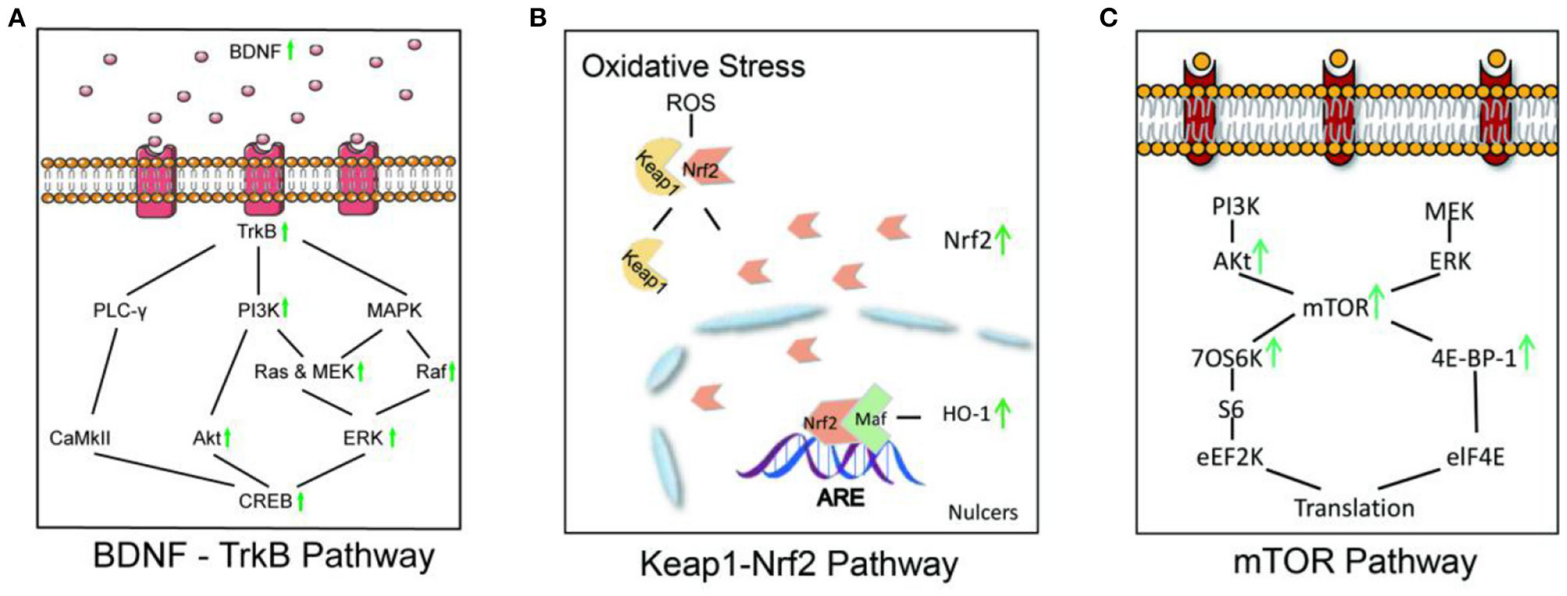
Acupuncture affects signaling pathways in PTSD model animals. **(A)** In the BDNF-TrkB signaling pathway, acupuncture increases the phosphorylation of the key downstream enzymes PI3K, Akt, MEK, Raf, ERK, and CREB by upregulating the expression of BDNF and TrkB. **(B)** In the Keap1-Nrf2 signaling pathway, acupuncture increased the expression of Nrf2 and HO-1, which improved hippocampal neurogenesis and ameliorated anxiety-like behaviors in PTSD model animals. **(C)** In the mTOR pathway, acupuncture improved PTSD symptoms by increasing p-mTOR, as well as the upstream protein, p-Akt, the downstream proteins, p-70S6K and p-4E-BP-1.

(1) BDNF-TrkB Pathway: Brain-derived neurotrophic factor (BDNF) is a basic protein that is found throughout the central nervous system. Mature BDNF first binds to tyrosine kinase receptor 2 (TRKB) to activate the downstream signaling molecules, MAPK and PLC-YPI3K, which promote neuronal survival and enhance neuronal mechanisms that regulate learning and memory. In adults, the BDNF-TrkB pathway has been identified as the primary regulator of fear circuit function and fear behavior expression (Ding, 2018). A similar study demonstrated that acupuncture improves fear gain, regression, and reconstruction in PTSD model animals by upregulating the expression levels of BDNF, TrkB, PI3K, p-Akt, p-MEK, and p-ERK (Ding, 2018; Li et al., 2020b). Furthermore, the cAMP-response element binding protein (CREB), a key protein in the BDNF-TrkB pathway, binds to postsynaptic density protein 95 (PSD95), a key protein at synapses, mediates changes in synaptic plasticity in neural structures, and participates in the acquisition, storage, and regression of fear memories. In a study, acupuncture was reported to improve CREB binding to PSD95, which regulates synaptic plasticity in PTSD model animals, thereby improving behavioral performance in PTSD model animals (Li et al., 2020a).

(2) Keap1-Nrf2 Pathway: The Kelch-like ECH-associated protein 1-NF-E2-related factor 2 (Keap1-Nrf2) pathway forms the major node of cellular and organismal defense against oxidative and electrophilic stresses of both exogenous and endogenous origins (Yamamoto et al., 2018). The Keap1-Nrf2 pathway controls the body's redox homeostasis. When the body is subjected to oxidative stress, the content of reactive oxygen species (ROS) increases, exceeding the ability of the cellular antioxidant defense system to clear them. Keap1 dissociates from Nrf2, and the dissociated Nrf2 translocates to the nucleus, activating the expression of phase II detoxification genes and associated antioxidant genes, such as haem oxygenase 1 (HO-1), to protect cells from oxidative stress (Zhang et al., 2021). A recent study demonstrated that acupuncture could upregulate the expression of Nrf2, HO-1, BDNF, and AMPK phosphorylation in PTSD model animals, whereas knocking out Nrf2 could reverse the protective effect of acupuncture, implying that acupuncture may play a role in the treatment of PTSD *via* the Keap1-Nrf2 pathway (Zhou et al., 2019).

(3) mTOR Pathway: The mammalian target of rapamycin (mTOR) is a serine/threonine kinase that regulates protein translation and has been linked to anxiety and depression (Cohen et al., 2004). By increasing the upstream protein ERK and activating the downstream proteins p70S6K and 4E-BP-1, mTOR activation effectively inhibits eukaryotic cell initiation factor 4E (EIF4E) and promotes protein translation (Zhuang et al., 2016). In a study, acupuncture improved PTSD symptoms by increasing p-mTOR, as well as the upstream protein p-Akt, the downstream protein p-70S6K, p-4E-BP-1, and p-CREB through the mTOR pathway (Oh et al., 2018) (Figure 4).

##### 3.3.2.4. Others

Zhang et al. (2019) indicated the regulatory effect of acupuncture on the blood oxygen level in the cerebral cortex. Zhou et al. (Lee and Pan, 2022) reported that acupuncture can reverse changes in lipid composition caused by the PTSD model (detail in Table 1).

**Table 1:** Effects of acupuncture on various parts of PTSD model animals.

**Part**	**Outcome raised**	**Outcome reduced**
Hippocampus	MR, Nrf2, SYN, HO-1, BDNF, TrkB, CREB, p-Akt, p-ERK, p-MEK, CB1R, DAGLα, AMPK	GR, Sirt1, Keap1, GRP78, lipocalin 2, Bcl-2 /Bax, CASPACE-12, nNOS, MAO-A, CHOP
Amygdala	BDNF, TrkB, p-MEK	TH, CRH, CRHR1
PFC	c-Fos, DAGLα, CB1R	GABAARal, IL-6
ACC	c-Fos	–
Hypothalamus	TNFα	IL-4
HPA axis	–	CRH, CORT, CRHR1, Serum cortisol, Serum corticosterone
others	5-HT, d-Hb, p-mTOR, p-p70S6K, p-4E-BP-1	t-Hb, HbO2, nNOS, TNF-a, IL-1β

## 4. Discussion

Patients with PTSD often suffer from a high symptom burden but low tolerance and compliance to pharmacotherapy. Additional regimens need to be explored. With the increasing availability of acupuncture worldwide, patients with PTSD are increasingly seeking and accepting acupuncture. Many studies have confirmed the effectiveness of acupuncture for PTSD (Yang and Zhang, 2012; Zhang, 2013; Jin et al., 2014, 2015; Zheng et al., 2014; Zhao et al., 2015; Han et al., 2016, 2017; Grant et al., 2018; Ding et al., 2020; Song et al., 2020, 2021; Hollifield et al., 2021; Kwon et al., 2021; Li et al., 2021). Because the number of studies in the last 10 years accounts for the vast majority and is limited by research technology, there are some differences in outcomes between the long-term research and the current research, so we searched various databases from 1 January 2012 to 27 November 2022. This review is divided into three sections as follows: a meta-analysis, an acupoint analysis, and mechanism research.

The most recent systematic review of acupuncture for PTSD was published in 2018 (Grant et al., 2018), which was 5 years ago and thus came before a number of recent randomized controlled trials of the treatment (Feng et al., 2019; Zhao et al., 2020). Of these two meta-analyses, only two articles (Wang et al., 2012; Engel et al., 2014) were identical due to differences in search time, search scope, and inclusion and exclusion criteria. The meta-analysis in 2018 (Grant et al., 2018) concluded that acupuncture treatment had a clear effect on improving symptoms in patients with PTSD, especially in the short-term and long-term effects of improving PTSD symptoms. Their outcomes of interest for meta-analyses were as follows: PTSD symptoms, health-related quality of life, functional status, depressive and anxiety symptoms, sleep quality, and adverse events. From the standpoint of how acupuncture affects the related scale score, we think we can use the CAPS, PCL-C, HAMD, HAMA, and SCL-90 somatization as outcome indicators to confirm whether acupuncture can successfully treat PTSD. Thus, we revised this meta-analysis on the use of acupuncture for PTSD. We concluded that acupuncture outperformed pharmacotherapy treatment in improving symptom scores of CAPS, HAMA, HAMD, PCL-C, and SCL-90 somatization for PTSD. At the same time, acupuncture is also better than psychotherapy in improving the scores of CAPS, PCL-C, and HAMD symptom scales. Moreover, according to the acupoint analysis of common acupoints, the top four clinical acupoints in PTSD treatment are GV20, GV24, GB20, and EX-HN1, while the top four animal acupoints are GV20, ST36, HT7, and GV24. GV20 is the most frequently used acupuncture point in clinical studies and animal studies, with a 78.6% application rate.

In mechanistic research, we investigated the potential effects of acupuncture on PTSD from three perspectives as follows: regulation of structure and components in several brain areas, neuroendocrine system regulation of PTSD, and signaling pathway involvement. According to the included studies, the hippocampus, amygdala, PFC, ACC, and hypothalamus are involved in the acupuncture treatment of PTSD. Most frequently, effects on the hippocampus have been reported. In PSTD, the hippocampus connects situational stimuli to aversive events and plays an important role in memory recall (Acheson et al., 2012). Thus, hippocampal dysfunction is regarded as an important factor for PTSD. Several studies included in this review have reported the potential impact of acupuncture on the hippocampus: acupuncture reduced microglial activation, downregulated lipocalin-2 in hippocampal astrocytes, and inhibited the overexpression of the key molecules GRP78, CHOP, Caspase-3, and Caspase-12 while reversing abnormal neural electrical activity in the CA1 and CA3 regions (Li, 2020; Liu et al., 2020; Chen et al., 2021; Sun Y. et al., 2022). In addition, acupuncture can increase amygdala and PFC, BDNF, increase PFC and ACC C-FOS expression, and decrease hypothalamic IL-4 and PFC IL-6 (Liu et al., 2019; Wei et al., 2019; Zhu and Lu, 2019; Chen et al., 2020; Hou et al., 2021; Yang et al., 2022). The pathological basis of PTSD in the neuroendocrine system is negative feedback inhibition of the HPA axis. Acupuncture regulates and repairs damaged neurons by decreasing CRH and CORT levels while increasing MR expression (Fang et al., 2012; Hou et al., 2013b; Wang, 2019; Lee and Pan, 2022; Zhu et al., 2022). Moreover, acupuncture also reduced the expression of nNOS and MAO-A while increasing the expression of DAGL and CB1R proteins, effectively alleviating the anxiety symptoms of PTSD models. In addition, acupuncture treats PTSD by acting on the BDNF-TrkB pathway, Keap1-Nrf2 pathway, mTOR pathway, and other signaling pathways (Ding, 2018; Oh et al., 2018; Zhou et al., 2019; Li et al., 2020a,b).

To the best of our knowledge, this is the most comprehensive review that summarizes the current progress on acupuncture treatment for PTSD. However, the following limitations and future perspectives should be considered. First, By searching relevant databases in Chinese and English, we included only eight articles involving 656 patients due to the small number of people with PTSD who use acupuncture. Due to the nature of acupuncture, achieving double-blindness in RCTs is difficult, and the methodological quality is not strictly evaluated. The included studies' sample size was small, we did not perform subgroup, and there could be publication bias. Second, five of the eight included studies had a high risk of blinding risk assessment of patients, which may be one of the sources of clinical heterogeneity. In future research, more rigorous, standardized, multicenter, large-sample, and high-quality RCTs guided by evidence-based medicine are needed. Third, the vast majority of the articles included in this review were conducted in China, so personnel may also have influenced the results. Fourth, it is challenging to discuss the particular effects of one treatment or one acupoint due to the methodological approach of the included studies. In the following studies, it is possible to focus on unique effects. For example, more groups should be used in the experimental design to show the effects and mechanisms of different acupoints. Fifth, there is a lack of agreement and unified standards for acupoint selection criteria. To better guide clinical practice and improve efficacy, it is recommended to build an expert consensus by combining Chinese medicine theory, expert experience, and evidence-based theories. Sixth, limited by current experimental techniques, the pathogenesis of PTSD and the influence of acupuncture cannot be fully revealed. Finally, acupoints, direction, depth, needle sensation, and other aspects of the treatment process are difficult to standardize due to the uniqueness of acupuncture. If multi-dimensional outcome indicators, standardized acupuncture operations, and long-term acupuncture effect records can be included in future experimental operations, the clinical feasibility of acupuncture in the treatment of PTSD can be better explored. At the same time, future research is urgently needed to investigate the mechanism of acupuncture treatment from more microscopic perspectives or in combination with tools such as functional magnetic resonance imaging or functional near-infrared spectroscopy.

In conclusion, many studies have confirmed that acupuncture could improve many symptoms in patients with PTSD by meta-analysis. We found that GV20 was the most frequently used acupoint in clinical studies and animal studies. Moreover, acupuncture's effect on PTSD may be mediated by the promotion of neuroprotection, neurogenesis, and synaptic plasticity in multiple brain areas, the regulation of stress responses in the neuroendocrine system, and signaling pathway involvement. This finding indicates that acupuncture has promising potential for treating PTSD. We anticipate that future studies will systematically elucidate the mechanism of acupuncture in the treatment of PTSD, allowing more PTSD patients to benefit from it.

## Author contributions

XT contributed to conceiving, writing the original draft, figure presentation, and editing. SL contributed to the literature search, data collection, and writing the original draft. DF and BL contributed to the literature search, data collection, and corrections. LY, LW, QX, and LL contributed to corrections and editing. NX contributed to conceiving, designing, editing, and supervising. All authors contributed to this article and approved this submitted version.

## References

[B1] Abanes J. J. Ridner S. H. Dietrich M. S. Hiers C. Rhoten B. (2022). Acupuncture for sleep disturbances in post-deployment military service members: a randomized controlled trial. Clin. Nurs. Res. 31, 239–250. 10.1177/10547738211030602 34229475

[B2] Acheson D. T. Gresack J. E. Risbrough V. B. (2012). Hippocampal dysfunction effects on context memory: possible etiology for posttraumatic stress disorder. Neuropharmacology 62, 674–685. 10.1016/j.neuropharm.2011.04.029 21596050 PMC3175276

[B3] Alvear M. P. (2021). Efficacy of Acupuncture for PTSD Among Bereaved Chilean People: A Randomized Controlled Study. Guangzhou University of Chinese Medicine.

[B4] Auxéméry Y. (2012). Posttraumatic stress disorder (PTSD) as a consequence of the interaction between an individual genetic susceptibility, a traumatogenic event and a social context. L'Encephale 38, 373–380. 10.1016/j.encep.2011.12.003 23062450

[B5] Battle D. E. (2013). Diagnostic and statistical manual of mental disorders (DSM). CoDAS 25, 191–192. 10.1590/s2317-17822013000200017 24413388

[B6] Benjet C. Bromet E. Karam E. G. Kessler R. C. McLaughlin K. A. Ruscio A. M.. (2016). The epidemiology of traumatic event exposure worldwide: results from the World Mental Health Survey Consortium. Psychol. Med. 46, 327–343. 10.1017/S0033291715001981 26511595 PMC4869975

[B7] Bremner J. D. Vythilingam M. Vermetten E. Southwick S. M. McGlashan T. Nazeer A.. (2003). MRI and PET study of deficits in hippocampal structure and function in women with childhood sexual abuse and posttraumatic stress disorder. Am. J. Psychiatry 160, 924–932. 10.1176/appi.ajp.160.5.924 12727697

[B8] Burbiel J. C. (2015). Primary prevention of posttraumatic stress disorder: drugs and implications. Milit. Med. Res. 2, 156–162. 10.1186/s40779-015-0053-2 26504586 PMC4620711

[B9] Canadian Psychiatric Association (2006). Clinical practice guidelines. Management of anxiety disorders. Can. J. Psychiatry 51(8 Suppl. 2), 9s−91s. 16933543

[B10] Chen Y. Xue F. Gu T. Wang S. Wang H. Peng Z. (2019). Effect of electroacupuncture pretreatment on anxiety-like behavior and expression of endogenous cannabinoidrelated genes in the prefrontal cortex of PTSD rats model. J. Neurosci. Ment. Health 19, 658–662. 10.3969/j.issn.1009-6574.2019.07.003

[B11] Chen Y. Xue S. Gu T. Wang H. Peng Z. (2020). Effect of early intervention with electroacupuncture on anxiety-like behavior and expression of BDNF, IL-1β and IL-6 in the prefrontal cortex of PTSD rats model. Prog. Modern Biomed. 20, 1619–1623. 10.13241/j.cnki.pmb.2020.09.004

[B12] Chen Y.-H. Xie S.-Y. Chen C.-W. Lu D.-Y. (2021). Electroacupuncture improves repeated social defeat stress-elicited social avoidance and anxiety-like behaviors by reducing Lipocalin-2 in the hippocampus. Mol. Brain 14:150. 10.1186/s13041-021-00860-0 34565419 PMC8474847

[B13] Cohen H. Zohar J. Matar M. A. Zeev K. Loewenthal U. Richter-Levin G. (2004). Setting apart the affected: the use of behavioral criteria in animal models of post traumatic stress disorder. Neuropsychopharmacology 29, 1962–1970. 10.1038/sj.npp.1300523 15257304

[B14] de Quervain D. Schwabe L. Roozendaal B. (2017). Stress, glucocorticoids and memory: implications for treating fear-related disorders. Nat. Rev. Neurosci. 18, 7–19. 10.1038/nrn.2016.155 27881856

[B15] Ding N. (2018). Based on the Amygdala BDNF-TrkB-ERK Signaling Pathway, the Effect of Electroacupuncture on Fear Memory in PTSD Rats Was Explored. Chengdu University of Traditional Chinese Medicine.

[B16] Ding N. Li L. Song K. Huang A. Zhang H. (2020). Efficacy and safety of acupuncture in treating post-traumatic stress disorder: a protocol for systematic review and meta-analysis. Medicine 99:e20700. 10.1097/MD.0000000000020700 32590744 PMC7328930

[B17] Dong Y. Gao W. (2012). The role and relationship of BCL-2, BAX, and Caspase-3 in apoptosis. Chinese J. Gerontol. 32, 4828–4830.

[B18] Engel C. C. Cordova E. H. Benedek D. M. Liu X. Gore K. L. Goertz C.. (2014). Randomized effectiveness trial of a brief course of acupuncture for posttraumatic stress disorder. Med Care. 52(12 Suppl. 5):S57–S64. 10.1097/MLR.0000000000000237 25397825

[B19] Errington-Evans N. (2012). Acupuncture for anxiety. CNS Neurosci. Therap. 18, 277–284. 10.1111/j.1755-5949.2011.00254.x 22070429 PMC6493505

[B20] Fang Y. Cai D. Zhou Q. Yu S. Peng X. Zheng Z. (2012). Comparative research on intervention in anxiety behavior of PTSD—·like rats by electro·acupuncture and repetitive transcranial m agnetic stimulation and serum corticosterone. J. Nanjing Univ. Tradit. Chinese Med. 28, 357–359. 10.3969/j.issn.1000-5005.2012.04.017

[B21] Feng B. Zhang Y. Luo L.-Y. Wu J.-Y. Yang S.-J. Zhang N.. (2019). Transcutaneous electrical acupoint stimulation for post-traumatic stress disorder: Assessor-blinded, randomized controlled study. Psychiatry Clin. Neurosci. 73, 179–186. 10.1111/pcn.12810 30565342

[B22] Grant S. Colaiaco B. Motala A. Shanman R. Sorbero M. Hempel S. (2018). Acupuncture for the treatment of adults with posttraumatic stress disorder: a systematic review and meta-analysis. J. Trauma Dissoc. 19, 39–58. 10.1080/15299732.2017.1289493 28151093

[B23] Gruol D. L. (2015). IL-6 regulation of synaptic function in the CNS. Neuropharmacology. 96(Pt A), 42–54. 10.1016/j.neuropharm.2014.10.023 25445486 PMC4446251

[B24] Guideline Development Panel for the Treatment of PTSD in Adults A. P. A.. (2019). Summary of the clinical practice guideline for the treatment of posttraumatic stress disorder (PTSD) in adults. Am. Psychol. 74, 596–607. 10.1037/amp0000473 31305099

[B25] Han Y. (2017). Intervention of “Liver Thinning” Acupuncture on Neurobehavioral and Cognitive Dysfunction in PTSD Model Rats. Gansu University of Traditional Chinese Medicine.

[B26] Han Y. Zhang Y. Yan X. (2016). Research progress on the mechanism of acupuncture in post-traumatic stress disorder. Chinese J. Inform. Tradit. Chinese Med. 4, 130–133. 10.3969/j.issn.1005-5304.2016.01.03426790233

[B27] Han Y. Zhang Y. Yan X. (2017). A meta-analysis of acupuncture for post-traumatic stress disorder. J. Gansu Coll. Tradit. Chinese Med. 34:69–74. 10.16841/j.issn1003-8450.2017.01.2132590744

[B28] Hollifield M. (2011). Acupuncture for posttraumatic stress disorder: conceptual, clinical, and biological data support further research. CNS Neurosci. Therap. 17, 769–779. 10.1111/j.1755-5949.2011.00241.x 22070661 PMC6493831

[B29] Hollifield M. Hsiao A. F. Carrick K. Gory Munoz A. Calloway T. Cocozza K.. (2021). Acupuncture for combat post-traumatic stress disorder: trial development and methodological approach for a randomized controlled clinical trial. Trials 22:594. 10.1186/s13063-021-05394-3 34488824 PMC8419889

[B30] Hou L. Liu C. Xiong K. (2013a). Effects of electroacupuncture on hippocampal nNOS expression in rats of post-traumatic stress disorder model. Chinese Acupunct. 33, 632–636. 10.13703/j.0255-2930.2013.07.019 24032201

[B31] Hou L. Liu S. Xiong K. (2013b). Effects of electroacupuncture intervention on behavior changes and hippocampal glucocorticoid receptor and mineralocorticoid receptor expression levels in post-traumatic stress disorder rats. Acupunct. Stud. 2:140–5. 10.13702/j.1000-0607.2013.02.010 23819217

[B32] Hou Y. Chen M. Wang C. Liu L. Mao H. Qu X.. (2021). Electroacupuncture attenuates anxiety-like behaviors in a rat model of post-traumatic stress disorder: the role of the ventromedial prefrontal cortex. Front. Neurosci. 15:159. 10.3389/fnins.2021.690159 34248490 PMC8264195

[B33] Jatzko A. Vogler C. Demirakca T. Ruf M. Malchow B. Falkai P.. (2013). Pattern and volume of the anterior cingulate cortex in chronic posttraumatic stress disorder (PTSD). Eur. Arch. Psychiatry Clin. Neurosci. 263, 585–592. 10.1007/s00406-013-0408-1 23613000

[B34] Ji Q. Li Z. Tang Y. Mo Y. Yao H. S G. (2013). Effect of electroacupuncture intervention on behavioral changes and hippocampal excitatory amino acid transporter mRNA expression in depression rats. Acupunct. Stud. 38, 202–207. 10.13702/j.1000-0607.2013.03.006 24006665

[B35] Jin R. Li J. Zheng C. Wang J. Zhang H. (2015). Progress of research on mechanisms of acupuncture intervention of post- traumatic stress disorder. Lishizhen Med. Mater. Med. Res. 26, 184–186. 10.3969/j.issn.1008-0805.2015.01.07526790233

[B36] Jin R. Li X. Zheng C. Wang J. Zhang H. (2014). Research progress of acupuncture treatment of post-traumatic stress disorder. China J. Tradit. Chinese Med. Pharmacy 29, 2883–2885.

[B37] Jin Z. Zhang W. Luo F. Zhang k Zhang L. Zeng Z.. (2001). Functional magnetic resonance imaging of the human brain's response to electrical stimulation at different frequency acupuncture points. Acta Physiol. Sin. 275–280.

[B38] King Heather C. Spence Dennis L. Hickey Anita H. Sargent P. Elesh R. Connelly Cynthia D. (2015). Auricular acupuncture for sleep disturbance in veterans with post-traumatic stress disorder: a feasibility study. Milit. Med. 180, 582–590. 10.7205/MILMED-D-14-00451 25939115

[B39] Koenen K. C. Ratanatharathorn A. Ng L. McLaughlin K. A. Bromet E. J. Stein D. J.. (2017). Posttraumatic stress disorder in the World Mental Health Surveys. Psychol. Med. 47, 2260–2274. 10.1017/S0033291717000708 28385165 PMC6034513

[B40] Kwon C.-Y. Lee B. Kim S.-H. (2021). Efficacy and underlying mechanism of acupuncture in the treatment of posttraumatic stress disorder: a systematic review of animal studies. J. Clin. Med. 10:8575. 10.3390/jcm10081575 33917977 PMC8068330

[B41] Lee B. Pan W. (2022). Neuroprotective effect of acupuncture against single prolonged stress-induced memory impairments and inflammation in rat brain via modulation of brain-derived neurotrophic factor expression. Evid. Based Complement. Altern. Med. 2022:4430484. 10.1155/2022/4430484 35251208 PMC8890831

[B42] Lee M. Y. Lee B. H. Kim H. Y. Yang C. H. (2021). Bidirectional role of acupuncture in the treatment of drug addiction. Neurosci. Biobehav. Rev. 126, 382–397. 10.1016/j.neubiorev.2021.04.004 33839169

[B43] Li B. (2012). Functional Magnetic Resonance Studies of Electroacupuncture in the Treatment of Post-Traumatic Stress Disorder. Chengdu University of Chinese Medicine.

[B44] Li F. Zhou C. Peng Z. Xue S. (2017). The influence of electroacupuncture (EA) preconditioning on anxiety-likebehavior and the expression of Sirt1/MAO-A in the hippocampusof PTSD rats. Prog. Modern Biomed. 17, 6839–6843. 10.13241/j.cnki.pmb.2017.35.009

[B45] Li M. Li K. Ding N. Xie Y.-Q. Niu K. Zhang H. (2020a). Effect of electroacupuncture on expression of CREB and its ability to bind to synaptic proteins in amygdala and hippocampus of rats with post-traumatic stress disorder. Acupuncture research. 45, 517–523. 10.13702/j.1000-0607.190709 32705823

[B46] Li M. Xie Y. Niu K. Li K. (2020b). Electroacupuncture ameliorates post-traumatic stress disorder in rats via a mechanism involving the BDNF-TrkB signaling pathway. Cell. Mol. Biol. 66, 165–170. 10.14715/cmb/2020.66.3.26 32538765

[B47] Li X. (2018). To Study the Effect of “Anshen Xingnao Tiaoshen” Electroacupuncture on Learning and Memory and the Expression of Hippocampal Synaptic Plasticity Related Proteins in PTSD Rats. Chengdu University of Chinese Medicine.

[B48] Li X. Quan M. Gao Z. Shi Y. (2007). Research progress on the mechanism of hippocampal volume change in traumatic stress disorder. Pract. Gen. Pract. 354–355. 10.16766/j.cnki.issn.1674-4152.2007.04.05727885969

[B49] Li X. Sun Y. Lu J. Jiang H. L. Tu Y. (2021). Progress of researches on acupuncture and moxibustion for treating post-traumatic stress disorder in the past five years. Acupunct. Stud. 46, 439–444. 10.13702/j.1000-0607.200654 34085470

[B50] Li Y. (2014). Effects of Electricity on PTSD Rat Behavior and Related Brain Region PKC. Heilongjiang University of Traditional Chinese Medicine.

[B51] Li Y. (2020). Effect of Acupuncture on Levels of Hippocampal Microglia in Single Prolongated Stressed Rats. Beijing University of Chinese Medicine.

[B52] Li Y. Zhao G. (2014). Effect of electroacupuncture on body weight and spatial learning and memory in PTSD rats. Heilongjiang Med. J. 38, 233–235. 10.3969/j.issn.1004-5775.2014.03.001

[B53] Liberzon I. Krstov M. Young E. A. (1997). Stress-restress: effects on ACTH and fast feedback. Psychoneuroendocrinology 22, 443–453. 10.1016/S0306-4530(97)00044-9 9364622

[B54] Liu L. Liu H. Hou Y. Shen J. Qu X. Liu S. (2019). Temporal effect of electroacupuncture on anxiety-like behaviors and c-Fos expression in the anterior cingulate cortex in a rat model of post-traumatic stress disorder. Neurosci. Lett. 711:134432. 10.1016/j.neulet.2019.134432 31419458

[B55] Liu Q. Wang L. Qi M. Yu H. Zhang G. (2020). Effects of electricity on learning and memory and Bcl-2/Bax expression in hippocampal CA1 region in PTSD-like rats. Acta Univ. Med. Anhui. 55, 340–344. 10.19405/j.cnki.issn1000-1492.2020.03.004

[B56] Lu J. Shi Y. Jin Z. Tu Y. (2003). A comparative study of the antidepressant effect of electricity at different frequencies on model rats. J. Beijing Univ. Chinese Med. 83–85.

[B57] Lu J. Wang W. Yuan Q. Zhang A. Chen M. Chen H.. (2016). Clinical study of applying Anshen Tongdu Kaiqiao acupuncture method in the treatment of post-traumatic stress disorde. J. Sichuan Tradit. Chinese Med. 34, 173–175.

[B58] Lu L. Zhang Y. Tang X. Ge S. Wen H. Zeng J.. (2022). Evidence on acupuncture therapies is underused in clinical practice and health policy. BMJ 376:e067475. 10.1136/bmj-2021-067475 35217525 PMC8868048

[B59] Lv T. Wu Y. (2022). Research progress in the mechanism of acupuncture improving hippocampal injury in post-traumatic stress disorder. Chinese J. Tradit. Chinese Med. 42:147–153. 10.13193/j.issn.1673-7717.2023.03.032

[B60] Michopoulos V. Rothbaum A. O. Jovanovic T. Almli L. M. Bradley B. Rothbaum B. O.. (2015). Association of CRP genetic variation and CRP level with elevated PTSD symptoms and physiological responses in a civilian population with high levels of trauma. Am. J. Psychiatry 172, 353–362. 10.1176/appi.ajp.2014.14020263 25827033 PMC4440454

[B61] Moiraghi C. Poli P. Piscitelli A. (2019). An observational study on acupuncture for earthquake-related post-traumatic stress disorder: the experience of the lombard association of medical acupuncturists/acupuncture in the world, in Amatrice, Central Italy. Med. Acupunct. 31, 116–122. 10.1089/acu.2018.1329 31031878 PMC6484341

[B62] Oh J. Y. Kim Y. K. Kim S. N. Lee B. Jang J. H. Kwon S.. (2018). Acupuncture modulates stress response by the mTOR signaling pathway in a rat post-traumatic stress disorder model. Sci. Rep. 8:11864. 10.1038/s41598-018-30337-5 30089868 PMC6082850

[B63] Pitman R. K. Rasmusson A. M. Koenen K. C. Shin L. M. Orr S. P. Gilbertson M. W.. (2012). Biological studies of post-traumatic stress disorder. Nat. Rev. Neurosci. 13, 769–787. 10.1038/nrn3339 23047775 PMC4951157

[B64] Prisco M. K. Jecmen M. C. Bloeser K. J. McCarron K. K. Akhter J. E. Duncan A. D.. (2013). Group auricular acupuncture for PTSD-related insomnia in veterans: a randomized trial. Med. Acupunct. 25, 407–422. 10.1089/acu.2013.0989

[B65] Shi T. Lu Z. Xiong L. (2017). Study on the effect mechanism of electroacupuncture at different frequencies on the diagnosis and treatment of central nervous system diseases. Chinese J. Neurosurg. Dis. Res. 16, 92–94.

[B66] Shin L. M. Orr S. P. Carson M. A. Rauch S. L. Macklin M. L. Lasko N. B.. (2004). Regional cerebral blood flow in the amygdala and medial prefrontal cortex during traumatic imagery in male and female Vietnam veterans with PTSD. Arch. Gen. Psychiatry 61, 168–176. 10.1001/archpsyc.61.2.168 14757593

[B67] Song K. Wang Y. T. Xiong F. J. Huang A. L. Zhang H. (2022). Effects of electroacupuncture on behaviors and expressions of SYN and PSD95 in hippocampus of rats with post-traumatic stress disorder. World J. Acupunct. 33, 135–141. 10.1016/j.wjam.2022.07.006

[B68] Song K. Zhang H. Xiong F. Huang A. (2020). Advances in neurobiology of acupuncture in the treatment of PTSD. World Chinese Med. 15, 3876–3880. 10.3969/j.issn.1673-7202.2020.24.030

[B69] Song K. Zhang H. Xiong F. Huang A. (2021). Law and actuality of acupuncture for post-traumatic stress disorder. J. Chengdu Univ. Chinese Med. 44:93–97. 10.13593/j.cnki.51-1501/r.2021.03.093

[B70] Spoont M. (2015). Jama patient page. Posttraumatic stress disorder (PTSD). JAMA 314:532. 10.1001/jama.2015.8109 26241611

[B71] Steenkamp M. M. Litz B. T. Hoge C. W. Marmar C. R. (2015). Psychotherapy for military-related PTSD: a review of randomized clinical trials. JAMA 314, 489–500. 10.1001/jama.2015.8370 26241600

[B72] Sun L. Yong Y. Wei P. Wang Y. Li H. Zhou Y.. (2022). Electroacupuncture ameliorates postoperative cognitive dysfunction and associated neuroinflammation via NLRP3 signal inhibition in aged mice. CNS Neurosci. Therap. 28, 390–400. 10.1111/cns.13784 34951130 PMC8841296

[B73] Sun Y. Li X. Shao R. Lu J. Tu Y. (2022). Effect of acupuncture on endoplasmic reticulum stress-related factors in hippocampus of posttraumatic stress disorder rats. Acupunct. Res. 47, 224–230. 10.13702/j.1000-0607.20210718 35319839

[B74] Takahashi Y. Okada T. (2011). Involvement of the nitric oxide cascade in melatonin-induced inhibition of long-term potentiation at hippocampal CA1 synapses. Neurosci. Res. 69, 1–7. 10.1016/j.neures.2010.09.004 20875465

[B75] Ursano R. J. Bell C. Eth S. Friedman M. Norwood A. Pfefferbaum B.. (2004). Practice guideline for the treatment of patients with acute stress disorder and posttraumatic stress disorder. Am. J. Psychiatry 161(11 Suppl.), 3–31. 15617511

[B76] Velazquez F. N. Caputto B. L. Boussin F. D. (2015). c-Fos importance for brain development. Aging 7, 1028–1029. 10.18632/aging.100862 26684501 PMC4712328

[B77] Wang C. (2019). The Mechanisms Underlying the Involvement of Glucocorticoid Receptor in the Electroacupuncture Regulating the Posttraumatic Stress Disorder. Shanghai University of Traditional Chinese Medicine.

[B78] Wang Y. (2013). Electroacupuncture for Treatment of Post-traumatic Stress Disorder Using Regional Homogeneity. Chengdu University of Chinese Medicine.

[B79] Wang Y. Hu Y.-P. Wang W. C. Pang R. Z Zhang A. R. (2012). Clinical studies on treatment of earthquake-caused posttraumatic stress disorder using electroacupuncture. Evid. Based Complement. Altern. Med. 2012:431279. 10.1155/2012/431279 23049609 PMC3462425

[B80] Wang Z. Zhou P. Tan Q. (2015). Efficacy of electroacupuncture combined with paroxetine in the treatment of post-traumatic stress disorder. J. Psychiatry. 326–328. 10.3969/j.issn.2095-9346.2015.05.002

[B81] Watkins L. E. Sprang K. R. Rothbaum B. O. (2018). Treating PTSD: a review of evidence-based psychotherapy interventions. Front. Behav. Neurosci. 12:258. 10.3389/fnbeh.2018.00258 30450043 PMC6224348

[B82] Webler R. D. Fulton S. Perera T. D. Coplan J. D. (2019). Maturational phase of hippocampal neurogenesis and cognitive flexibility. Neurosci. Lett. 711:134414. 10.1016/j.neulet.2019.134414 31430544

[B83] Wei K. Huang C. Chen X. Cao H. (2019). Effects of electrical acupuncturing in Baihui acupoint on sleep phases of post-traumatic stress disorder rats and the mechanisms. Shaanxi J. Tradit. Chinese Med. 40, 1333–1335. 10.3969/j.issn.1000-7369.2019.10.004

[B84] Wei Z. Li L. A Bajor L. Ticlea A. N. N.. (2013). Guidelines for clinical pharmacotherapy for post-traumatic stress disorder - psychopharmacology of the Harvard Southshore Program (PAPHSS). Int. J. Psychiatry 40, 49–53. 10.13479/j.cnki.jip.2013.01.017

[B85] Wu H. Zhao Y. Hou Y. Yang X. (2013). Acupuncture combined with repetitive transcranial magnetic stimulation in the treatment of post-traumatic stress disorder. J. Brain Neurol. Dis. 21.

[B86] Xie K. Tang G. Zahn H. Yang K. Zhao J. (2015). Effect of electroacupuncture on locus nNOS expression in rats of post-traumatic stress disorder model. Med. Theory Pract. 28:2421–2423. 10.19381/j.issn.1001-7585.2015.18.001

[B87] Xue F. Xue S. Liu L. Sang H.-F. Ma Q Tan Q. R.. (2019). Early intervention with electroacupuncture prevents PTSD-like behaviors in rats through enhancing hippocampal endocannabinoid signaling. Prog. Neuro-Psychopharmacol. Biol. Psychiatry 93, 171–181. 10.1016/j.pnpbp.2019.03.018 30946940

[B88] Yamamoto M. Kensler T. W. Motohashi H. (2018). The KEAP1-NRF2 system: a thiol-based sensor-effector apparatus for maintaining redox homeostasis. Physiol. Rev. 98, 1169–1203. 10.1152/physrev.00023.2017 29717933 PMC9762786

[B89] Yang S. Zhang H. (2012). “Post-traumatic stress disorder and its effect and prospect of acupuncture treatment,” in Proceedings of the 2012 Sichuan Acupuncture and Moxibustion Association Annual Conference (Xichang), 356–357.

[B90] Yang Y. Wu C. Qin S. Lu J. Mao H. Qu X.. (2022). Effects of CO, laser stimulation at acupoint ST36 on behaviors and infralimbic cortex c- Fos expression in rat model of post-traumatic stress disorder. Shanghai J. Tradit. Chinese Med. 56, 79–84. 10.16305/j.1007-1334.2022.2111045

[B91] Yao Y. Fang Y. (2019). Research progress on the role of ketamine and endocannabinoid system in rapid antidepressant. J. Shanghai Jiao Tong Univ. 39, 428–431.

[B92] Yu C. Tian L. Zhang S. Yao Y. (2019). Effect of acupuncture and moxibustion on the spatial and temporal patterns of abnormal neuronal information in hippocampus of rats with sleep disorder induced by post-traumatic stress disorder and the ultrastructure of damaged neurons. J. Sichuan Tradit. Chinese Med. 37, 29–32.

[B93] Yue N. Li B. Yang L. Han Q. Q. Huang H. J. Wang Y. L.. (2018). Electro-acupuncture alleviates chronic unpredictable stress-induced depressive- and anxiety-like behavior and hippocampal neuroinflammation in rat model of depression. Front. Mol. Neurosci. 11:149. 10.3389/fnmol.2018.00149 29946236 PMC6007169

[B94] Zhang Q. Liu J. Duan H. Li R. Peng W. Wu C. (2021). Activation of Nrf2/HO-1 signaling: an important molecular mechanism of herbal medicine in the treatment of atherosclerosis via the protection of vascular endothelial cells from oxidative stress. J. Adv. Res. 34, 43–63. 10.1016/j.jare.2021.06.023 35024180 PMC8655139

[B95] Zhang Y. Han Y. Zhao Z. Yan X. Yang Y. (2019). Effect of acupuncture on blood oxygen concentration in brain of rats with post-traumatic stress disorder based on functional near-infrared spectroscopy. Acupunct. Tuina Med. 17:9–15. 10.1007/s11726-019-1083-1

[B96] Zhang Y. Qiu C. Tang X. Zhang W. (2015). Neurobiocharacterization of sleep disorders in patients with post-traumatic stress disorder. Int. J. Psychiatry 42, 123–125.36056716

[B97] Zhang Y. Zheng H. Zhao J. Liu H. (2017). Clinical manifestations of adverse reactions to SSRI and SNRI class antidepressants and their care. J. Clin. Ration. Drug Use 10, 127–128. 10.15887/j.cnki.13-1389/r.2017.22.074

[B98] Zhang Z. (2013). A Meta-Analysis of the Efficacy of Acupuncture in the Treatment of Post-Traumatic Stress Disorder. Chengdu University of Chinese Medicine.

[B99] Zhao G. Li X. Li M. Wang H. Wang L. Chang Y.. (2020). Clinical observation of female post-traumatic stress disorder treated by electroacupuncture by tonifying kidney and dredging du meridian method. Liaoning J. Tradit. Chinese Med. 47, 175–177. 10.13192/j.issn.1000-1719.2020.02.054

[B100] Zhao G. Liu M. Guo S. Zhang Y. Li Y. Xu W.. (2014). Observation of the efficacy of electroacupuncture Baihui-large vertebral points on post-traumatic stress disorder. Clin. J. Acupunct.

[B101] Zhao Y. Han Y. Zhang Y. Zhu T. Ma Z. Zhao Z.. (2018). Acupuncture intervention improves behavior reactions and learning-memory ability in post-traumatic stress disorder rats. Acupunct. Res. 43, 562–566. 10.13702/j.1000-0607.170652 30232864

[B102] Zhao Z. Zhang A. Zhang Y. Zhu T. Zhao Y. Liu A.. (2018). Effects of dispersing liver and regulating spirit acupuncture therapy on spatial and temporal patterns of neural coding in hippocampal CA1, CA3 regions of rats with PTSD. China J. Tradit. Chinese Med. Pharm. 33, 3895–3900.

[B103] Zhao Z. Zhang W. Xing J. Yan X. (2015). Modern research progress regarding effect mechanism of acupuncture on post-traumatic stress disorder. Chinese Acupunct. Moxibust. 35, 1085–1088. 10.13703/j.0255-2930.2015.10.034 26790233

[B104] Zhao Z. Zhao Y. Zhu T. Xing J. Pu X. Zhang Y.. (2019). Effects of acupuncture on neuro-electrophysiological activities in hippocampal CA1 and CA3 areas of rats with post-traumatic stress disorder. Acupunct. Tuina Med. 17, 67–73. 10.1007/s11726-019-1095-x

[B105] Zhao Z. Q. (2008). Neural mechanism underlying acupuncture analgesia. Prog. Neurobiol. 85, 355–375. 10.1016/j.pneurobio.2008.05.004 18582529

[B106] Zheng C. (2015). Study on the Influence of Electrical Targeting of Amygdala Functional Connection Network in PTSD Patients Based on rs-fMRI Technology: Chengdu University of Chinese Medicine.

[B107] Zheng C. Tan L. Zhou T. Zhang H. (2015). Effects of electroacupuncture on resting-state encephalic functional connectivity network in patients with PTSD. Chinese Acupunct. Moxibust. 35, 469–473. 10.13703/j.0255-2930.2015.05.017 26255522

[B108] Zheng C. Zhou T. Jin R. Li X. Wang J. Zhang H. (2014). Research progress of having used acupuncture to treat post-traumatic stress disorder in the rencent five years of domestic and overseas. J. Sichuan Tradit. Chinese Med. 32, 189–190.

[B109] Zhou C. Xue F. Shi Q. Xue S.-S. Zhang T. Ma X.-X.. (2022). The impact of electroacupuncture early intervention on the brain lipidome in a mouse model of post-traumatic stress disorder. Front. Mol. Neurosci. 15:812479. 10.3389/fnmol.2022.812479 35221914 PMC8866946

[B110] Zhou C. H. Xue F. Xue S.-s. Sang H.-f. Liu L. Wang Y.. (2019). Electroacupuncture pretreatment ameliorates PTSD-like behaviors in rats by enhancing hippocampal neurogenesis via the Keap1/Nrf2 antioxidant signaling pathway. Front. Cell. Neurosci. 13:275. 10.3389/fncel.2019.00275 31293390 PMC6598452

[B111] Zhou P. Zhang Y. Tan Q. (2015). Effects of electric acupuncture combined with sertraline in the treatment of post-traumatic stress disorder. Sichuan Ment. Health. 7, 504–506. 10.11886/j.issn.1007-3256.2015.06.00630565342

[B112] Zhou Q. Liu K. Han S. Gao X. (2022). Role of animal experiment in acupuncture translational medicine. Chinese Acupunct. 42, 1339–1343. 10.13703/j.0255-2930.20220803-k0003 36484184

[B113] Zhu B. (2021). On the acupoint and its specificity. Chinese Acupunct. 41, 943–950. 10.13703/j.0255-2930.20210701-k0002 34491640

[B114] Zhu J. Wang C. Wang Y. Guo C. Lu P. Mou F.. (2022). Electroacupuncture alleviates anxiety and modulates amygdala CRH/CRHR1 signaling in single prolonged stress mice. Acupunct. Med. 40, 369–378. 10.1177/09645284211056352 35044840

[B115] Zhu X. Lu Y. (2019). Electroacupuncture inhibits increase of TH and decrease of BDNF in the amygdala of PTSD rats. Acta Acad. Med. Wannan. 38, 115–119. 10.3969/j.issn.1002-0217.2019.02

[B116] Zhu X. Wang W. Zong Y. Yang H. Lu Y. (2016). Electroacupuncture inhibits decrease of BDNF and increase ofGABAARal in the medial prefrontal cortex of PTSD rats. Chinese J. Histochem. Cytochem. 25, 54–58. 10.16705/j.cnki.1004-1850.2016.01.010

[B117] Zhuang F. Li M. Gao X. Wang Y. Wang D. Ma X.. (2016). The antidepressant-like effect of alarin is related to TrkB-mTOR signaling and synaptic plasticity. Behav. Brain Res. 313, 158–171. 10.1016/j.bbr.2016.06.057 27374162

