# Supplementary material for: Efficacy and underlying mechanisms of acupuncture therapy for PTSD: evidence from animal and clinical studies
Source: Front Behav Neurosci. 2023 May 2;17:1163718. doi: 10.3389/fnbeh.2023.1163718 (PMC10187757; doi:10.3389/fnbeh.2023.1163718)
Supplement: Supplementary file 1 [file Table_1.pdf]

Supplementary Table (S. Table1-5)

S. Table 1. Search Terms Used in Pubmed.

	Searches	Details
#1	"Posttraumatic stress disorder"[All Fields] OR "PTSD"[All Fields] OR "Post-Traumatic Neuroses"[All Fields]	39118
#2	"Acupuncture and moxibustion"[All Fields] OR "Acupuncture"[All Fields] OR "Moxibustion"[All Fields] OR "Pharmacopuncture"[All Fields] OR "plum-blossom needle"[All Fields] OR "elongated needle"[All Fields] OR "abdominal acupuncture"[All Fields] OR "Electroacupuncture"[All Fields] OR "Auricular needle"[All Fields] OR "Scalp acupuncture"[All Fields] OR "Wrist ankle needle"[All Fields] OR "Superficial needling"[All Fields] OR "Warm needling"[All Fields] OR "Fire needling"[All Fields] OR "Acupoint injection"[All Fields] OR "Auricular point"[All Fields] OR "Acupoint stimulation"[All Fields] OR "needle"[All Fields]	189667
#3	"2012/01/01"[Date - Publication] : "2022/11/27"[Date - Publication]	13316371
#4	#1 AND #2 AND #3	107

S. Table 2. Characteristics of included Meta-analysis studies.

Author	*N* Acupuncture group /*N* Control group	Acupuncture group interventions	Control group interventions	Acupuncture Point	Frequency/Hz	Course of Treatment	Outcome Indicator
Wang2012	63/64	EA	Paroxetine	GV20,EX-HN1,GV24,GB20	100	30min each time,1 time/2d,continuous 12w	CAPS,HAMD,HAMA,TESS
Wu2013	15/15	MA+Guipi Decoction+High Frequency rTMS	Sertraline +High Frequency rTMS	Top midline, parietal sideline, apical slope first line,top slope second line, GB20,LI14,LI11,TE5,ST36,KI1	-	30min each time,2 time/d,continuous 2W	PCL-C
Zhao2014a	32/30	EA	Paroxetine	GC20,GV4	100	30min each time,6 time/w,continuous 8w	ETI,SCL-90,TESS
Engel2014b	28/27	MA+Medications + psychotherapy	Medications + psychotherapy	BL13, BL14,BL15,BL17,B20,LR3, LI4, HT5,HT7, PC6,KI3,KI9,CV4,CV15,GV24,TE4,GV29	-	60min each time,2 time/w,continuous 4w	PCL-C,CAPS,NRS,BDI-II,SF-36 MCS,SF-36 PCS
Zhou2015	24/23	EA+Sertraline	Sertraline	GV20,GV29,PC6,ST36	1.3-1.7	30min each time,5 time/w,continuous 4w	PCL,HAMA
Lu2016	15/15	EA	Sertraline	GV20,EX-HN1,GV24,GB20	100	30min each time,1 time/d,continuous 10d	PCL-M,HAMD, HAMA
Feng2019	120/120	TEAS+CBT/TEAS+CBT+Sertraline	Sertraline /CBT	PC6	50	1 time/W,continuous 12W	CAPS,PCL-C,HAMD,TESS
Zhao2020	33/32	EA	Paroxetine	GV20,GV4,BL23,BL52	100	30min each time,6 time/w,continuous 8w	SCL-90,Cortisol,Estradiol

S. Table 3. Characteristics of included clinical acupoint analysis studies.

Year	Acupuncture group interventions	Acupuncture Point	Frequency/Hz	Outcome Indicator
Yu2012	EA	GV20,EX-HN1,GV24,GB20	100	30min each time, 1 time/2d, continuous 12w
Li2012	EA	GV20,EX-HN1,GV24,GB20	100	30min each time, 3 times/w, continuous 12w
Wu2013	scalp acupuncture+MA	Top midline, parietal sideline, apical slope first line,top slope second line, GB20,LI14,LI11,TE5,ST36,KI1	-	30min each time, 2 times/d, continuous 2w
Michelle2013	ear acupuncture	Sympathetic, liver, shen men, kidney	-	45min each time, 2 times/w, continuous 8w
Wang2013	EA	GV20,EX-HN1,GV24,GB20	50	60min each time, 3 times/w, continuous 12w
Zhao2014	EA	GV20,GV14	100	30min each time, 6 times/w, continuous 8w
Engel2014	MA+UPC	BL13,BL14,BL15,BL18,B20, LR3, LI4, HT5,HT7, PC6,KI3,KI9,CV4,CV15,GV24,TE4,GV24+	-	60min each time, 2 times/w, continuous 4w
Zhou2015	EA	GV20,GV24+,PC6,ST36	1.3-1.7	30min each time, 5 times /w, continuous 4w
CDR2015	ear acupuncture	Shen men, point zero, brain, thalamus point, pineal gland, master cerebral, insomnia points 1 and 2, kidney, heart, occiput, and forehead	-	30min each time, 3 times /w, continuous 3w
Zheng2015	MA	GV20,EX-HN1,GV24,GB20	-	30min each time, 3 times/w, continuous 12w
Wang2015	EA	GV20,GV24+,PC6,ST36	1.3-1.7	30min each time, 5 times /w continuous 4w
Tan2015	EA	GV20,EX-HN1,GV24,GB20	50	30min each time, 3 times/w, continuous 12w
Lu2016	EA	GV20,EX-HN1,GV24,GB20	100	30min each time, 1 time/d, continuous 10d
Feng2019	TEAS	PC6	50	1 time/w, continuous 12w
Carlo2019	MA	Kidney meridian,Large Intestine meridian,Spleen meridian, Gall Bladder meridian	-	20min each time, 4 times/d continuous 5w
Zhao2020	EA	GV20,GV14,BL52,BL23	100	30min each time, 6 times /w, continuous 8w
Michael2021	MA	LR3,PC6,HT7,ST36,SP6,GB20	-	60min each time, 2 times /w, continuous 12w
Mireya2021	MA	GV20,EX-HN1,BL13,BL15,BL18,BL20,BL23	-	30min each time, 2 times /w, continuous 5w
Jane2022	MA	GV20,GV24,HT7,LI4,LR3	-	30min each time, 1 time /w, continuous 8w

S. Table 4. Characteristics of included animal acupoint analysis studies.

Year	Acupuncture group interventions	Acupuncture Point	Frequency/Hz	Outcome Indicator
Fang2012	EA	GV1,GV20	0.25~0.42	15min/d, continuous 3d
Hou2013a	EA	ST36,GV20	2	30min/d, continuous 7d
Hou2013b	EA	GV20,ST36	2	30min/d, continuous 7d
Li2014a	EA	GV20,GV14	2	20min/d, continuous 10d
Li2014b	EA	GV20,GV14	50	20min/d, continuous 14d
Xie2015	EA	GV20,ST36	2	30min/d, continuous 7d
Zhu2016	EA	GV20,ST36	2	30min/d, continuous 21d
Li2017	EA	GV20	2~15	30min/d, continuous 7d
Han2017	MA	GV20,PC6,HT7,LR3	-	4min/d, continuous 5d
Zhao2018a	MA	GV20,PC6,HT7,LR3	-	4min/d, continuous 12d
Zhao2018b	MA	GV20,PC6,HT7,LR3	-	4min/d, continuous 14d
Ding2018	MA	GV20,GV24,BL23	2~100	20min/d, continuous 21d
Li2018	MA	GV20,GV24,BL23	2~100	30min/d, continuous 21d
Oh2018	MA	HT7,HT8	2	30s/d,continuous 4d
Zhou2019	EA	GV20	2~15	30min/d, continuous 7d
Xue2019	EA	GV20	2~15	30min/d, continuous 7d
Yu2019	MA	GV20,PC6,HT7,LR3	-	4min/d, continuous 7d
Zhang2019	MA	GV20,PC6,HT7,LR3	-	4min/d, continuous 12d
Zhu2019	EA	ST36,GV20	2	30min/d, continuous 21d
Wang2019	EA	ST36,GV20	2~15	30min/d, continuous 5d
Wei2019	EA	GV20	50	20min/d, continuous 7d
Chen2019	EA	GV20	2~15	30min/d, continuous 7d
Zhao2019	MA	GV20,PC6,HT7,LR3	-	4min/d, continuous 14d
Liu2019	EA	ST36	2	20min/d, continuous 7d
Li2020a	EA	GV20,GV24,BL23	2~15	20min/d, continuous 21d
Chen2020	EA	GV20	2~15	30min/d, continuous 7d
Li2020b	MA	GV20,GV24+	-	20min/d, continuous 15d
Li2020c	EA	GV20,GV24,BL23	2~100	20min/d, continuous 21d
Liu2020	EA	GV20,GV14,ST36	5~10	15min/d, continuous 14d
Yu2020	EA	ST36	2	30min/d, continuous 14d
Chen2021	EA	GV20,GV14	2	20min/d, continuous 5d
sun2021	MA	GV20,GV14	-	10min/d, continuous 7d
Yang2021	C02 laser stimulation	ST36	-	20min/d, continuous 7d
Zhou2022	EA	GV20	2~15	30min/d, continuous 7d
Jing2022	EA	ST36,GV20	2~15	30min/d, continuous 7d
Lee2022	MA	ST36,HT7	2	5min/d, continuous 21d
Song2022	EA	GV20,GV24,BL18,BL23	2~100	20min/d, continuous 21d

S. Table 5. Characteristics of included mechanism research studies.

Author	object	Molding method	Outcome Indicator	
			raised	reduced
Yang2012	SD rats	SPS	-	Serum corticosterone
Hou2013a	SD rats	SPS	MR	GR
Hou2013b	SD rats	SPS	-	nNOS mRNA
Li2014	SD rats	ESPS	-	-
Xie2015	SD rats	SPS	-	nNOS in locus coeruleus
Zhu2016	Wistar rats	SPS	BDNF	GABAARal
Li2017	SD rats	SPS	-	Sirt1 and MAO-A mRNA
Zhao2018a	SD rats	Shock and claustrophobia	-	-
Zhao2018b	SD rats	Shock and claustrophobia	Discharge frequency and concentration distribution frequency band	-
Oh2018	SD rats	SPS	p-Akt, p-mTOR, p-p70S6K, p-4E-BP-1,p-CREB	Serum corticosterone, Corticotropinreleasing factor
Ding2018	SD rats	SPS/SPS&S	BDNF,TrkB,p-ERK,p-MEK,PSD95,SYN1, GluR1	-
Zhou2019	SD rats	ESPS	Nrf2, HO-1, BDNF, AMPK	Keap1
Fen2019	SD rats	SPS	BDNF, DAGLα, CB1R, syn	-
Yu2019	Wistar rats	Shock and claustrophobia	Firing amplitude	-
Zhang2019	SD rats	Shock and claustrophobia	D-Hb	HbO2, T-Hb
Zhu2019	Wistar rats	SPS	BDNF	TH
Wang2019	C57BL/6 mice	SPS	Serum ACTH	GR
Wei2019	SD rats	SPS	TNFα	IL-4
Chen2019	SD rats	ESPS	CB1R and DAGLα proteins	-

Continued S. Table 5. Characteristics of included mechanism research studies.

Author	object	Molding method	Outcome Indicator	
			raised	reduced
Zhao2019	SD rats	Shock and claustrophobia	PSD concentration area	ISI
Liu2019	SD rats	SPS	c-Fos	Plasma corticosterone
Li2020a	SD rats	SPS	p-MEK, p-ERK1/2, PI3K, p-Akt, p-CREB, BDNF, TrkB mRNA, and the binding of CREB to the BDNF promoter region	-
Chen2020	SD rats	ESPS	BDNF	IL-6
Li2020b	SD rats	SPS	Number of cells	Microglia, IL-1β
Li2020c	SD rats	SPS	The binding ability of CRBE and CREB to PSD95 protein	-
Liu2020	SD rats	SPS	Hippocampal neurons	Bcl-2 /Bax
Hou2021	SD rats	MSPS	c-Fos	-
Chen2021	C57BL/6 mice	RSDS	-	lipocalin 2 expression, hippocampal astrocytes
Sun2022	SD rats	SPS	-	GRP78, CHOPmRNA, CASPACE-12
Yang2022	SD rats	SPS	c-Fos	Plasma corticosterone
Zhou2022	C57BL/6 mice	MSPS	SM, CL, ACCA, FA, Co, LPG	Cer, GM1, LPS, PE, TG
Zhu2022	C57BL/6 mice	SPS	-	CRH, CRHR1
Lee2022	SD rats	SPS	BDNF, TrkB	Serum cortisol, TNFα, COX-2
