# Supplementary material for: Efficacy and underlying mechanisms of acupuncture therapy for PTSD: evidence from animal and clinical studies
Source: Front Behav Neurosci. 2023 May 2;17:1163718. doi: 10.3389/fnbeh.2023.1163718 (PMC10187757; doi:10.3389/fnbeh.2023.1163718)
Supplement: Supplementary file 2 [file Image_1.PDF]

Supplementary Figures

S. Figure 1. Evaluation of the methodological quality of articles included in the meta-analysis

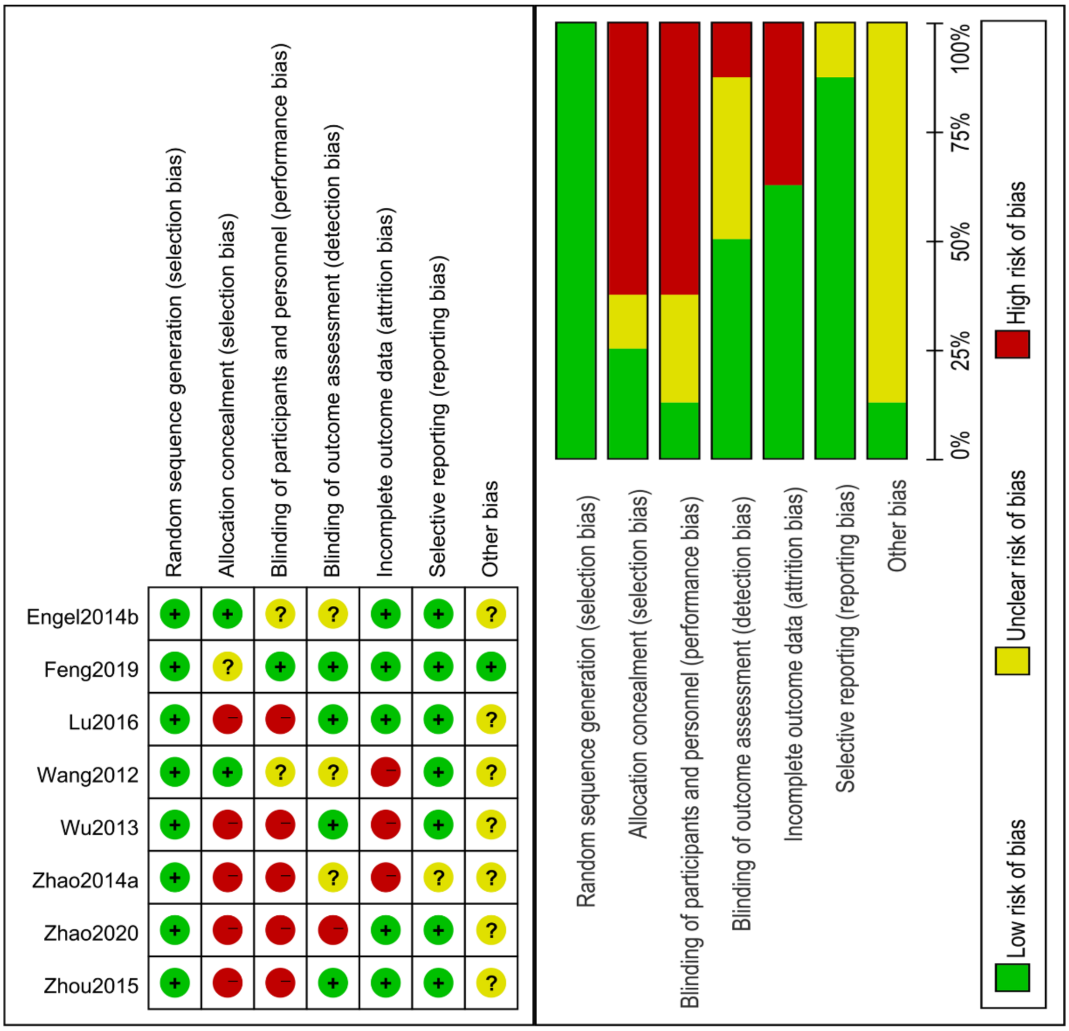

S. Figure 2. Sources of heterogeneity in HAMA meta-analysis

A

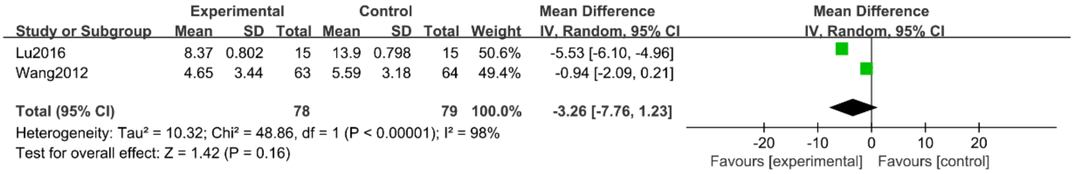

B

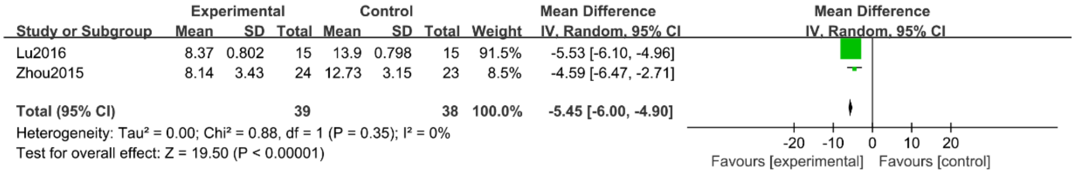

C

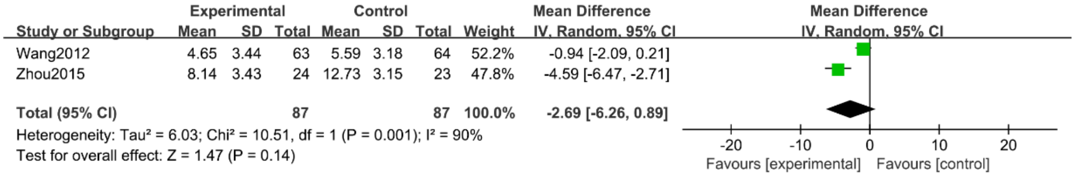

（A）Lu2016 and Wang2012 conducted a meta-analysis of the random effects model（B）Lu2016 and Zhou2015 conducted a meta-analysis of the random effects model（C）Wang2012 and Zhou2015 conducted a meta-analysis of the random effects model

S. Figure 3. Sources of heterogeneity in HAMD meta-analysis

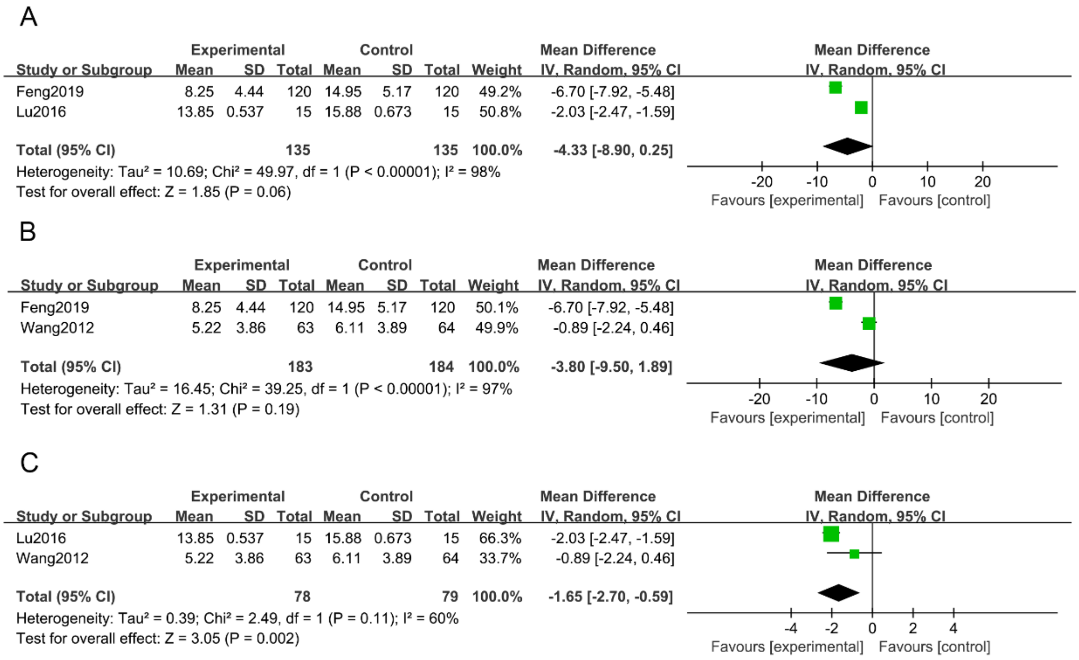

（A）Feng2019 and Lu2016 conducted a meta-analysis of the random effects model（B）Feng2019 and Wang2012 conducted a meta-analysis of the random effects model（C）Lu2016 and Wang2012 conducted a meta-analysis of the random effects model
